# Bismuth Silicate Catalyst for Efficient Electrocatalytic CO_2_ Reduction and Electrolyte‐Free Formic Acid Production

**DOI:** 10.1002/advs.202506034

**Published:** 2025-08-11

**Authors:** Ping Zhu, Xin‐Hao Cai, Cheng‐Cheng Huang, Ying Zhou, Na Chu, Zi‐Bo Jing, Wen‐Long Wang, Bilu Liu, Yong Jiang, Qian‐Yuan Wu

**Affiliations:** ^1^ Shenzhen Key Laboratory of Ecological Remediation and Carbon Sequestration Key Laboratory of Microorganism Application and Risk Control Ministry of Ecology and Environment State Key Laboratory of Regional Environment and Sustainability Institute of Environment and Ecology Shenzhen International Graduate School Tsinghua University Shenzhen 518055 P. R. China; ^2^ Key Laboratory of Microorganism Application and Risk Control of Shenzhen Guangdong Provincial Engineering Research Center for Urban Water Recycling and Environmental Safety Institute of Environment and Ecology Shenzhen International Graduate School Tsinghua University Shenzhen 518055 P. R. China; ^3^ Department of Materials Science and Technology University of Science and Technology of China Anhui 230026 P. R. China; ^4^ Fujian Key Laboratory of Pollution Control and Resource Reuse College of Environmental and Resource Sciences Fujian Normal University Fuzhou 350117 P. R. China; ^5^ Institute of Materials Research Tsinghua Shenzhen International Graduate School Tsinghua University Shenzhen 518055 P. R. China; ^6^ College of Resources and Environment Fujian Agriculture and Forestry University Fuzhou 350002 P. R. China

**Keywords:** Bi@Bi_2_O_2_CO_3_, bismuth silicate, electrocatalytic CO_2_ reduction, electrolyte‐free formic acid, reconstruction process, solid‐state electrolyte

## Abstract

The rising atmospheric CO_2_ levels pose significant environmental challenges. Electrocatalytic CO_2_ reduction offers a promising approach for converting CO_2_ into valuable chemicals such as formate or formic acid. However, the development of efficient electrocatalysts, a deeper mechanistic understanding, and the minimization of energy consumption during product purification remain critical challenges to practical carbon utilization. Here, layered Bi_2_SiO_5_ is designed as a pre‐catalyst, which undergoes electrochemical reconstruction into a Bi@Bi_2_O_2_CO_3_ composite. The catalyst achieves a Faradaic efficiency for formate of 95.8% at −1.06 V and maintains over 90% across a wide potential range, outperforming Bi_2_O_2_CO_3_ and Bi. In situ characterizations reveal that Bi_2_SiO_5_ converts to Bi_2_O_2_CO_3_ through anion exchange, followed by partial reduction to form Bi@Bi_2_O_2_CO_3_. Charge redistribution at the interface facilitates the proton‐coupled electron transfer of ^*^CO_2_ and desorption of ^*^HCOOH, thereby enhancing formate production, as supported by theoretical calculations. Furthermore, integrating the catalyst into an electrolytic cell containing solid‐state electrolytes enables the continuous production of electrolyte‐free formic acid, simplifying product separation and purification. This work provides insights for the development of practical carbon utilization.

## Graphical abstract

Layered Bi_2_SiO_5_ is designed that electrochemically reconstructs into a highly active Bi@Bi_2_O_2_CO_3_ catalyst for CO_2_‐to‐formate conversion. The system operates efficiently over a wide operating range. Coupling with solid‐state electrolyte system enables continuous production of electrolyte‐free formic acid, simplifying separation and reducing energy consumption. This work provides insights for the development of practical carbon utilization.

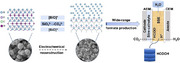

## 1 Introduction

With rapid global economic growth and increasing energy demand, carbon dioxide (CO_2_) emissions have risen significantly, resulting in severe environmental issues such as global warming, ocean acidification, and species extinction.^[^ ^1^, ^2^, ^3^ ^]^ Consequently, reducing the concentration of CO_2_ in the atmosphere has emerged as a critical global challenge. CO_2_ is a highly stable molecule that requires external energy for activation during reactions.^[^ ^4^, ^5^ ^]^ Electrocatalytic CO_2_ reduction (ECR), powered by renewable energy, presents a promising approach for converting CO_2_ into valuable chemical products due to its mild operational conditions, scalability, and controllable process.^[^ ^6^, ^7^, ^8^, ^9^ ^]^ The development of electrocatalysts with high activity and selectivity is essential for advancing ECR technology.

CO_2_ can be converted into a variety of products through different electron transfer pathways.^[^ ^10^ ^]^ Among these products, formate and formic acid are widely regarded as ideal hydrogen carriers and important chemical fuels for direct formate fuel cells.^[^ ^11^, ^12^ ^]^ However, achieving efficient production requires catalysts that optimize intermediate adsorption while suppressing side reactions. P‐block transition metal‐based catalysts, including bismuth, indium, lead, and tin, have been identified as the most effective electrocatalysts for ECR to formate due to their optimal capacity for intermediate adsorption.^[^ ^13^, ^14^, ^15^ ^]^ Notably,  bismuth‐based (Bi‐based) catalysts have garnered significant interest due to their low toxicity, high abundance, and cost‐effectiveness.^[^ ^16^, ^17^ ^]^

However, Bi‐based catalysts often undergo structural transformations during the electrochemical reduction process, making it difficult to understand their intrinsic catalytic mechanisms.^[^ ^18^, ^19^, ^20^ ^]^ To address this challenge, recent strategies have focused on pre‐designed structures that undergo controlled reconstruction during operation, allowing for the formation of active phases with enhanced catalytic performance.^[^ ^21^, ^22^, ^23^, ^24^, ^25^, ^26^ ^]^ Layered Aurivillius‐phase bismuth oxides, composed of alternating [BiO]^+^ slabs and interlayer [X]^−^ anions, provide an ideal platform for investigating electrochemical reconstruction and catalytic behavior due to their high specific surface area, excellent electrical conductivity, and tunable structure.^[^ ^27^, ^28^, ^29^, ^30^, ^31^, ^32^, ^33^ ^]^

Additionally, one of the primary challenges limiting the practical application of ECR is the separation of liquid‐phase products. In conventional electrolysis systems, such as H ‐cell and flow cell, the product is typically generated in aqueous electrolytes (e.g., KHCO_3_ or KOH). In such environments, the liquid‐phase products inevitably become mixed with ionic species including HCO_3_^−^ and K^+^, thereby complicating their direct utilization and downstream processing.^[^ ^34^, ^35^, ^36^, ^37^ ^]^ To obtain pure products, additional separation steps such as acidification, solvent extraction, and concentration are often required. These processes are energy‐intensive and significantly increase operational costs, thereby undermining the economic viability and scalability of ECR technologies.^[^ ^38^, ^39^, ^40^ ^]^ Simplifying or eliminating the separation step is therefore considered a critical strategy for advancing practical ECR systems. In this context, electrolysis systems based on solid‐state electrolytes (SSE) have attracted increasing attention. By replacing liquid electrolytes with solid ion‐conducting media, SSE cell enable the direct production of high‐purity, electrolyte‐free liquid products and offer a promising route to circumvent the need for post‐electrolysis separation.^[^ ^41^, ^42^, ^43^, ^44^ ^]^

In this study, the layered bismuth silicate (Bi_2_SiO_5_) was utilized as a pre‐catalyst and electrochemically transformed into post‐electrolysis sample (BOS_R_), a composite of Bi clusters and Bi_2_O_2_CO_3_ (Bi@Bi_2_O_2_CO_3_), to enhance the ECR performance. The catalyst exhibited excellent formate selectivity in an H‐cell, maintaining a formate Faradaic efficiency (FE_formate_) above 90% across a wide potential range (−0.86 V to −1.26 V), with a peak value of 95.8% at −1.06 V. This performance surpasses that of most reported Bi‐based catalysts, including pure Bi_2_O_2_CO_3_ and Bi. Ex situ and in situ characterizations revealed that Bi_2_SiO_5_ underwent anion exchange to form Bi_2_O_2_CO_3_, followed by partial reduction to generate Bi@Bi_2_O_2_CO_3_. Theoretical calculations indicated that this unique structure facilitates charge redistribution at the interface, thereby modulating intermediate adsorption and promoting the hydrogenation of ^*^CO_2_ and the desorption of ^*^HCOOH. Furthermore, an electrolytic cell incorporating a solid‐state electrolyte was constructed, in which the catalyst demonstrated continuous generation of electrolyte‐free formic acid solution.

## 2 Results and Discussion

### 2.1 Synthesis and Characterization of Catalysts

**Figure 1:**
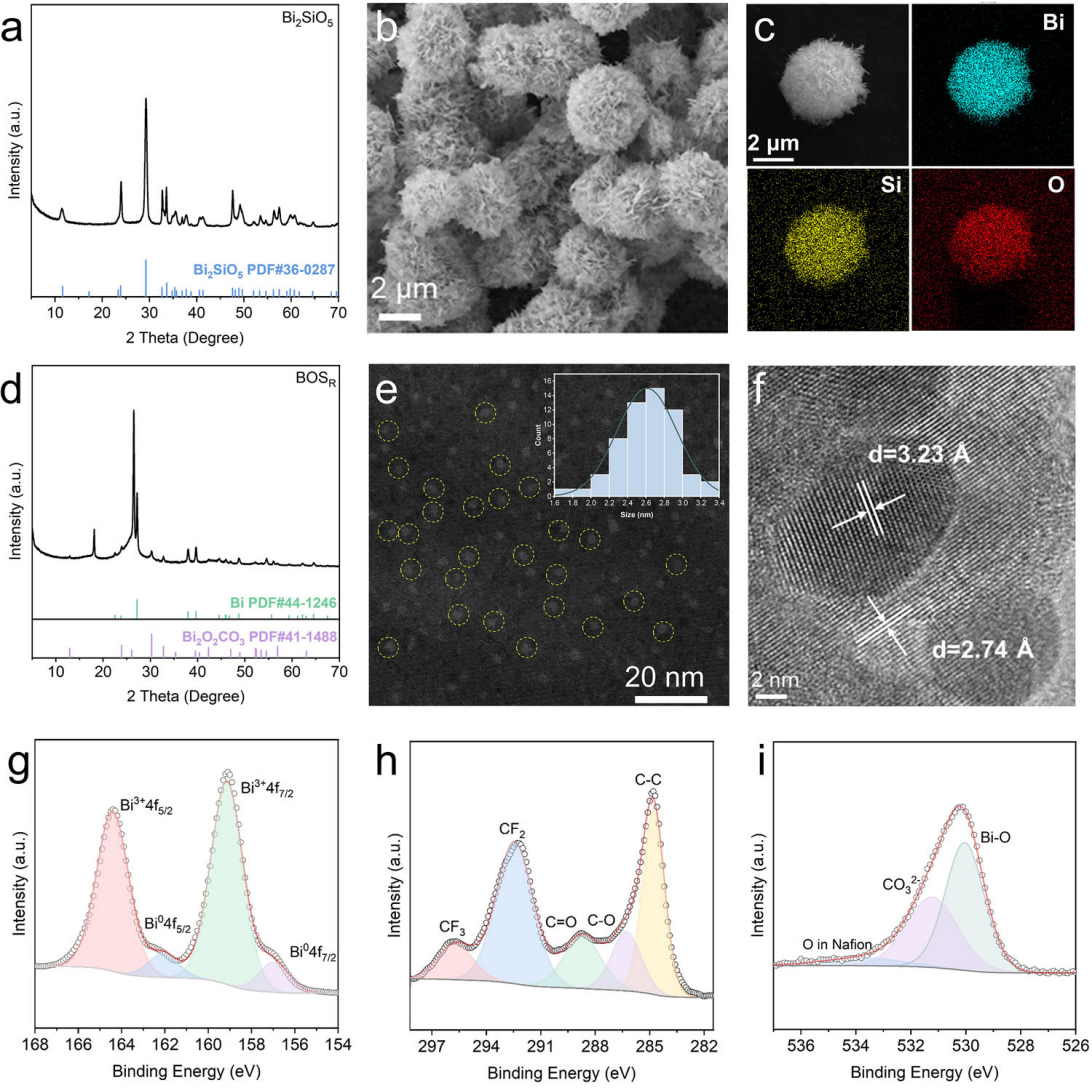
Structural characterizations of catalysts. a) XRD pattern, b) SEM image, c) EDS elemental mapping images of Bi_2_SiO_5_. d) XRD pattern, e) HAADF‐STEM image, f) HRTEM image, and g) Bi 4f, h) C 1s, i) O 1s XPS spectrum of BOS_R_.

Bismuth silicate (Bi_2_SiO_5_) was synthesized through a simple solvothermal method from Bi (NO_3_)_3_.5H_2_O and Na_2_SiO_3_.9H_2_O (Figure S1, Supporting Information). X‐ray diffraction (XRD) analysis (**Figure** **1**a) confirmed the successful formation of phase‐pure Bi_2_SiO_5_, with diffraction peaks matching well with the standard pattern (PDF#36‐0287). Scanning electron microscopy (SEM, Figure 1b; Figure S2, Supporting Information) revealed uniform microspheres assembled from stacked 2D nanosheets. High‐resolution transmission electron microscopy (HRTEM, Figure S3, Supporting Information) revealed clear lattice fringes with a spacing of 0.31 nm corresponding to the (311) plane. Energydispersive X‐ray spectroscopy (EDS, Figure 1c) demonstrated the uniform distribution of Bi, Si, and O elements. X‐ray photoelectron spectroscopy (XPS, Figure S4, Supporting Information) further confirmed the presence of Bi, Si, and O, with the Bi 4f spectrum exhibiting characteristic peaks of Bi^3+^ at 159.17 and 164.48 eV. The Si 2p signal at 102.15 eV, between those of Si and SiO_2_, indicated the presence of an oxygen‐angle sharing tetrahedron (SiO_3_)^2−^ structure. The O 1s spectrum can be deconvoluted into three components at 530.08, 531.82, and 533.21 eV, corresponding to Si─O, Bi─O, and adsorbed oxygen, respectively. In addition, Fourier‐transform infrared spectroscopy (FTIR, Figure S5, Supporting Information) confirmed the presence of Bi─O, Si─O, and Bi─O─Si linkages. These results collectively verify the successful synthesis of phase‐pure, well‐defined Bi_2_SiO_5_ microspheres.

The Bi_2_SiO_5_ precursor immobilized onto commercial carbon paper (1.0 mg cm^−2^) was electrochemically transformed at −1.06 V versus reversible hydrogen electrode (RHE) for 1 h in CO_2_‐saturated 0.5 m KHCO_3_ solution to yield the post‐electrolysis sample (BOS_R_). XRD patterns (Figure 1d) revealed the disappearance of all Bi_2_SiO_5_ diffraction peaks in BOS_R_, replaced by distinct peaks corresponding to hexagonal Bi (PDF#44‐1246) and tetragonal Bi_2_O_2_CO_3_ (PDF#41‐1488), along with signals from the carbon paper substrate. Morphological analysis by SEM revealed a 2D nanosheet morphology for the BOS_R_ catalyst (Figure S6, Supporting Information). High‐angle annular dark‐field scanning transmission electron microscopy (HAADF‐STEM) demonstrated that Bi clusters,^[^ ^45^ ^]^ with an average size of 2.61 nm, are uniformly distributed on the surface of Bi_2_O_2_CO_3_ (Figure 1e). HRTEM image (Figure 1f) revealed well‐defined two‐phase interfaces, with lattice spacings of 0.323 and 0.274 nm, corresponding to the (012) plane of metallic Bi and the (110) plane of Bi_2_O_2_CO_3_. The survey spectrum (Figure S7, Supporting Information) detected Bi, O, C, F, and K, with Si absent, suggesting the full transformation of Bi_2_SiO_5_. The Bi 4f spectrum (Figure 1g) exhibited doublets corresponding to Bi^3+^ (159.14, 164.41 eV) and Bi^0^ (156.97, 162.07 eV). Deconvolution of the C 1s spectrum (Figure 1h) identified C─C (284.80 eV), C─O (286.28 eV), C═O in CO_3_^2−^ (288.71 eV), and CF_2_/CF_3_ from Nafion binder (292.44, 295.70 eV).^[^ ^46^ ^]^ The O 1s spectrum (Figure 1i) exhibited peaks for Bi─O lattice oxygen (530.04 eV), C═O in carbonate (531.22 eV), and oxygen in Nafion (533.72 eV).^[^ ^47^, ^48^ ^]^ The observed 1.75 eV decrease in Bi─O binding energy suggested interfacial charge redistribution from metallic Bi to Bi^3+^ in Bi_2_O_2_CO_3_.^[^ ^39^, ^49^, ^50^ ^]^ Therefore, Bi_2_SiO_5_ underwent electrochemical reconstruction to form an active Bi@Bi_2_O_2_CO_3_ composite, which plays a crucial role in enhancing catalytic activity and selectivity for CO_2_ electroreduction.

### 2.2 Investigation of the Reconstruction Process

**Figure 2:**
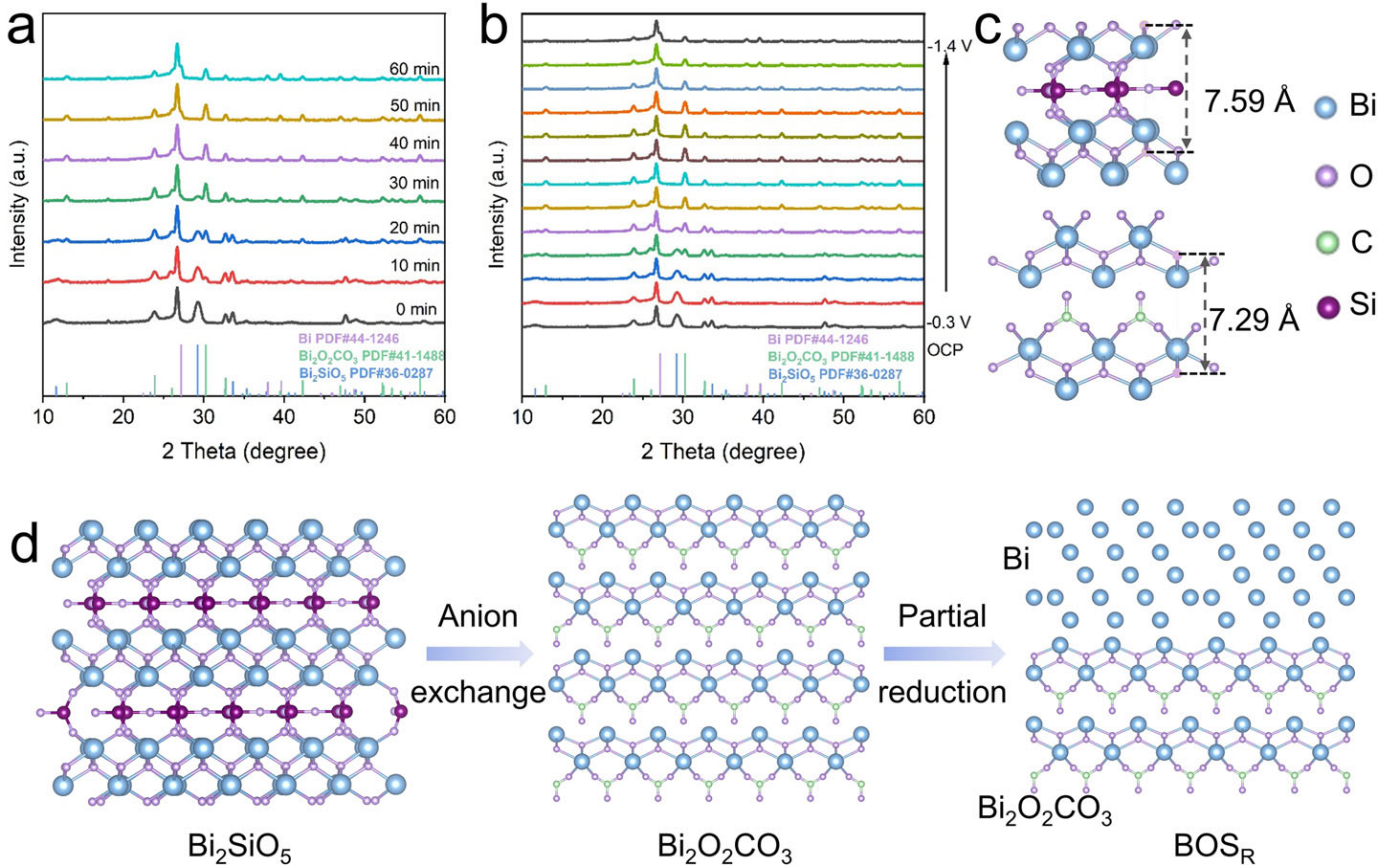
Reconstruction process of catalysts. a) Time‐resolved and b) potential‐resolved in situ XRD patterns of Bi_2_SiO_5_ in CO_2_‐saturated 0.5 m KHCO_3_ solution. c) Schematic diagram of the crystal structures of Bi_2_SiO_5_ (top) and Bi_2_O_2_CO_3_ (bottom). d) Illustration of the structural evolution from Bi_2_SiO_5_ to BOS_R_.

To gain more comprehensive insights into the reconstruction process of Bi_2_SiO_5_ under electrochemical conditions, time‐resolved in situ XRD patterns analyses were systematically conducted during its electrochemical conversion in CO_2_‐saturated 0.5 m KHCO_3_ solution (**Figure** **2**a). The diffraction peaks corresponding to Bi_2_SiO_5_ gradually diminished over time, while those of Bi_2_O_2_CO_3_ emerged and intensified. Eventually, the Bi_2_SiO_5_ signals disappeared completely, indicating full conversion into Bi_2_O_2_CO_3_. As the electrolysis time further increased, additional peaks corresponding to metallic Bi appeared, suggesting that part of the Bi_2_O_2_CO_3_ underwent further reduction to form Bi^0^, resulting in the formation of Bi@Bi_2_O_2_CO_3_. This transformation pathway was confirmed by the potential‐resolved in situ XRD measurements (Figure 2b).

To clarify the origin of the carbon species in Bi_2_O_2_CO_3_, control experiments combined with XRD phase analysis were conducted under different gas and electrolyte environments (Figure S8, Supporting Information). Bi_2_O_2_CO_3_ formed in 0.5 m KHCO_3_ under N_2_ atmosphere but was absent in 0.5 m K_2_SO_4_ under CO_2_ atmosphere, indicating that the carbonate originated from the electrolyte rather than the gaseous CO_2_, consistent with previous studies.^[^ ^51^, ^52^, ^53^, ^54^, ^55^ ^]^ In addition, when Bi_2_SiO_5_ was simply immersed in 0.5 m KHCO_3_ without any applied potential, no significant phase changes were detected, confirming that the observed phase transformation is driven by cathodic polarization during electrochemical conversion process  (Figure S9, Supporting Information).

The phase transformation from Bi_2_SiO_5_ to Bi_2_O_2_CO_3_ can be structurally rationalized by their shared layered architectures, both comprising alternating positively charged [BiO]^+^ slabs and negatively charged SiO_3_^2−^ or CO_3_^2−^ anionic layers. As shown in Figure 2c, Bi_2_SiO_5_ has a larger interlayer spacing of the (Bi_2_O_2_)^2+^ layers (7.59 Å), compared to Bi_2_O_2_CO_3_ (7.29 Å), which facilitates the diffusion of electrolyte ions and allows the insertion and replacement of intercalated SiO_3_^2−^ by CO_3_^2−^ anions.^[^ ^56^, ^57^ ^]^ Therefore, under cathodic conditions, Bi_2_SiO_5_ underwent a complete anion exchange transformation into Bi_2_O_2_CO_3_, which is subsequently partially reduced to metallic Bi, resulting in the formation of a Bi@Bi_2_O_2_CO_3_ composite, as illustrated in Figure 2d.

### 2.3 Electrochemical CO_2_ Reduction Performance

**Figure 3:**
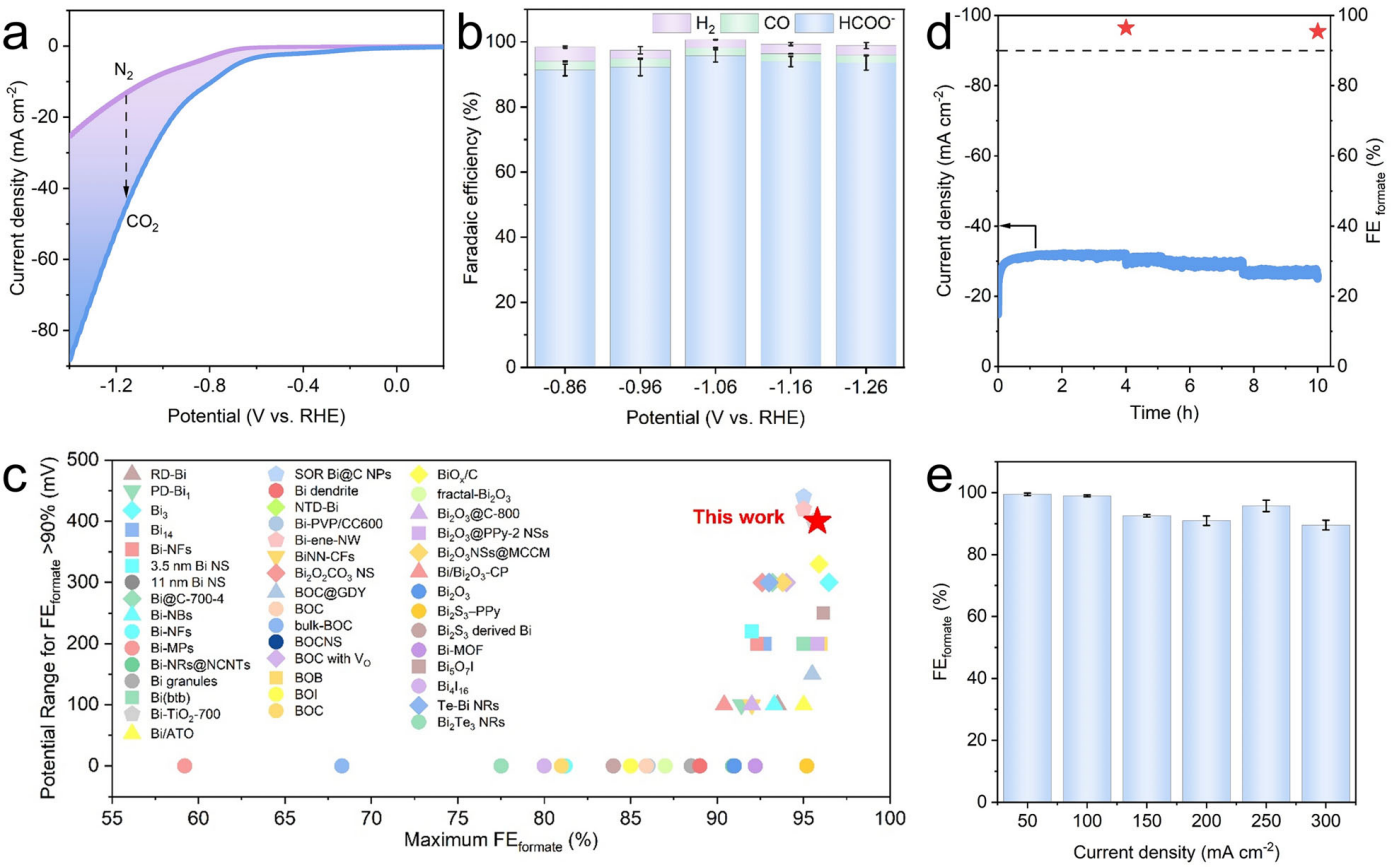
ECR performance of BOS_R_ in H‐cell and flow cell. a) LSV curves in CO_2_ and N_2_‐saturated 0.5 m KHCO_3_. b) Faradaic efficiencies of various products in the H‐cell. c) Comparison of ECR performance with reported Bi‐based electrocatalysts. d) Stability test of BOS_R_ in the H‐cell. e) FE_formate_ at different current densities in the flow cell.

The electrochemical CO_2_ reduction (ECR) performance of the BOS_R_ catalyst was systematically evaluated using a gas‐tight three‐electrode H‐cell with 0.5 m KHCO_3_ (Figure S10, Supporting Information). Linear sweep voltammetry (LSV, **Figure** **3**a) revealed significantly higher current density and a more positive onset potential under CO_2_‐saturated electrolyte compared to N_2_‐saturated electrolyte, indicating facilitated CO_2_ reduction reaction and suppressed side hydrogen evolution reaction. Subsequent constant potential electrolysis at selected applied potentials ranging from −0.86 to −1.26 V versus RHE showed that formate was the dominant product with only trace amounts of H_2_ and CO were detected (Figure 3b; Figures S11 and S12, Supporting Information). Specifically, the Faradaic efficiency of CO remained ≈2%, while H_2_ fluctuated slightly between 2% and 5%. A maximum formate Faradaic efficiency (FE_formate_) of 95.8% was achieved at −1.06 V versus RHE, and values consistently exceeded 91% across the entire potential range. This performance is significantly superior to that of most Bi‐based catalysts (Figure 3c; Table S1, Supporting Information). Moreover, the BOS_R_ catalyst demonstrated excellent electrochemical stability, maintaining steady current density and high formate selectivity during extended electrolysis, confirming its operational durability (Figure 3d). In a flow cell with 1.0 m KOH electrolyte (Figure S13, Supporting Information), the catalyst maintained high formate selectivity across a broad range of current densities from 50 to 300 mA cm^−2^(Figure 3e), highlighting its strong potential for practical CO_2_ electroreduction.

**Figure 4:**
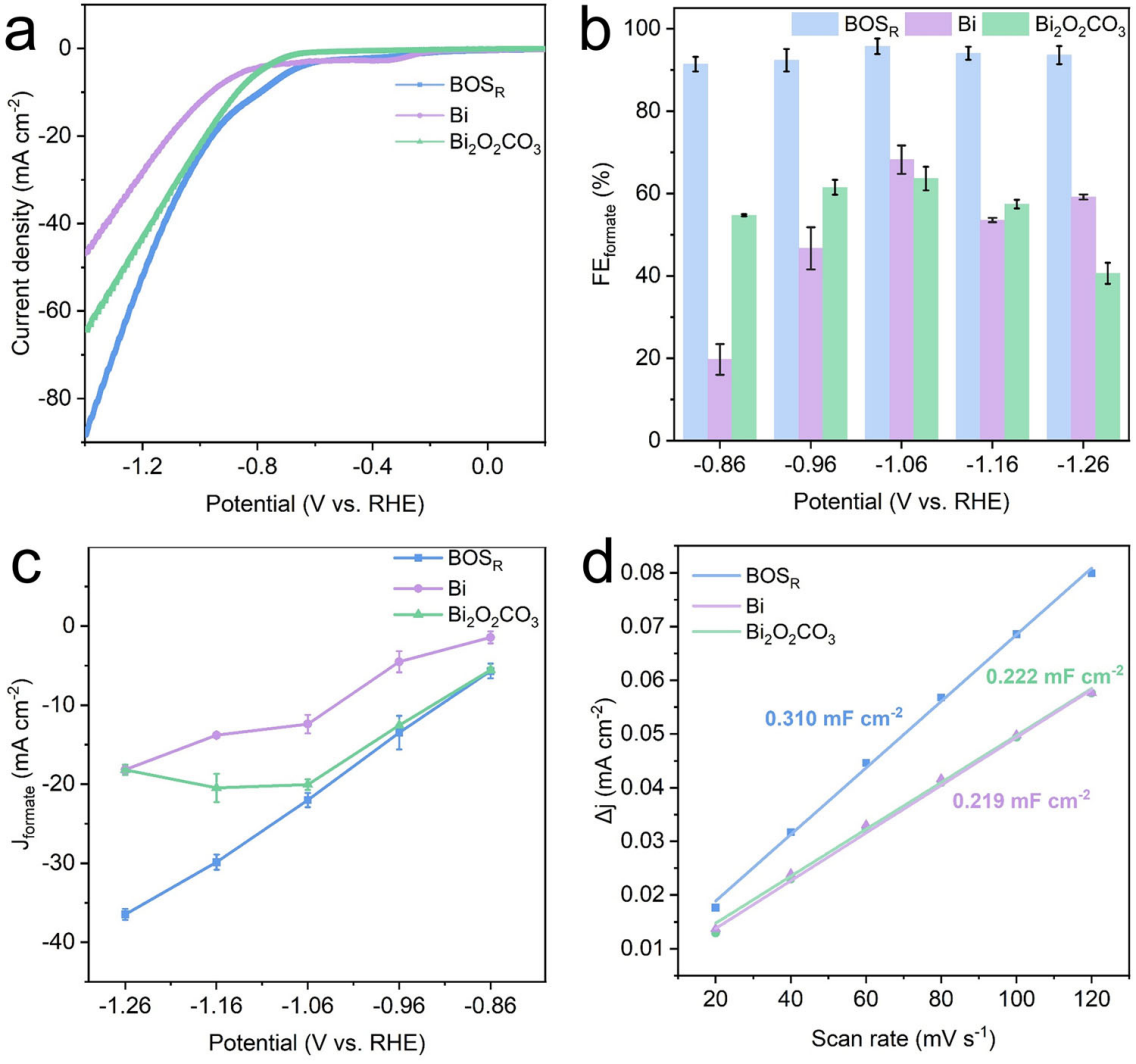
Comparison of ECR performance of BOS_R_, Bi, and Bi_2_O_2_CO_3_. a) LSV curves in CO_2_‐saturated 0.5 m KHCO_3_. b) FE_formate_ at different applied potentials. c) J_formate_ at different applied potentials. d) Current density versus scan rate plots.

To validate the electrocatalytic superiority of the BOS_R_ catalyst, comparative studies were conducted using pure Bi and Bi_2_O_2_CO_3_ as control catalysts. As shown in **Figure** **4**a, the catalyst exhibited higher current density and a more positive onset potential than Bi and Bi_2_O_2_CO_3_. It also achieved the highest FE_formate_ and the largest partial formate current densities (J_formate_) across all applied potentials (Figure 4b,c). The electrochemically active surface area (ECSA) was estimated by measuring the double‐layer capacitance (C_dl_) using cyclic voltammetry at scan rates ranging from 20 to 120 mV s^−1^ (Figure S14, Supporting Information). As shown in Figure 4d, BOS_R_ exhibits a C_dl_ of 0.310 mF cm^−2^, corresponding to an ECSA of 14.7 cm^2^, surpassing Bi_2_O_2_CO_3_ (10.6 cm^2^) and Bi (10.4 cm^2^), thereby offering more accessible active sites that contribute to its superior CO_2_ reduction activity.

### 2.4 Mechanistic Investigations

**Figure 5:**
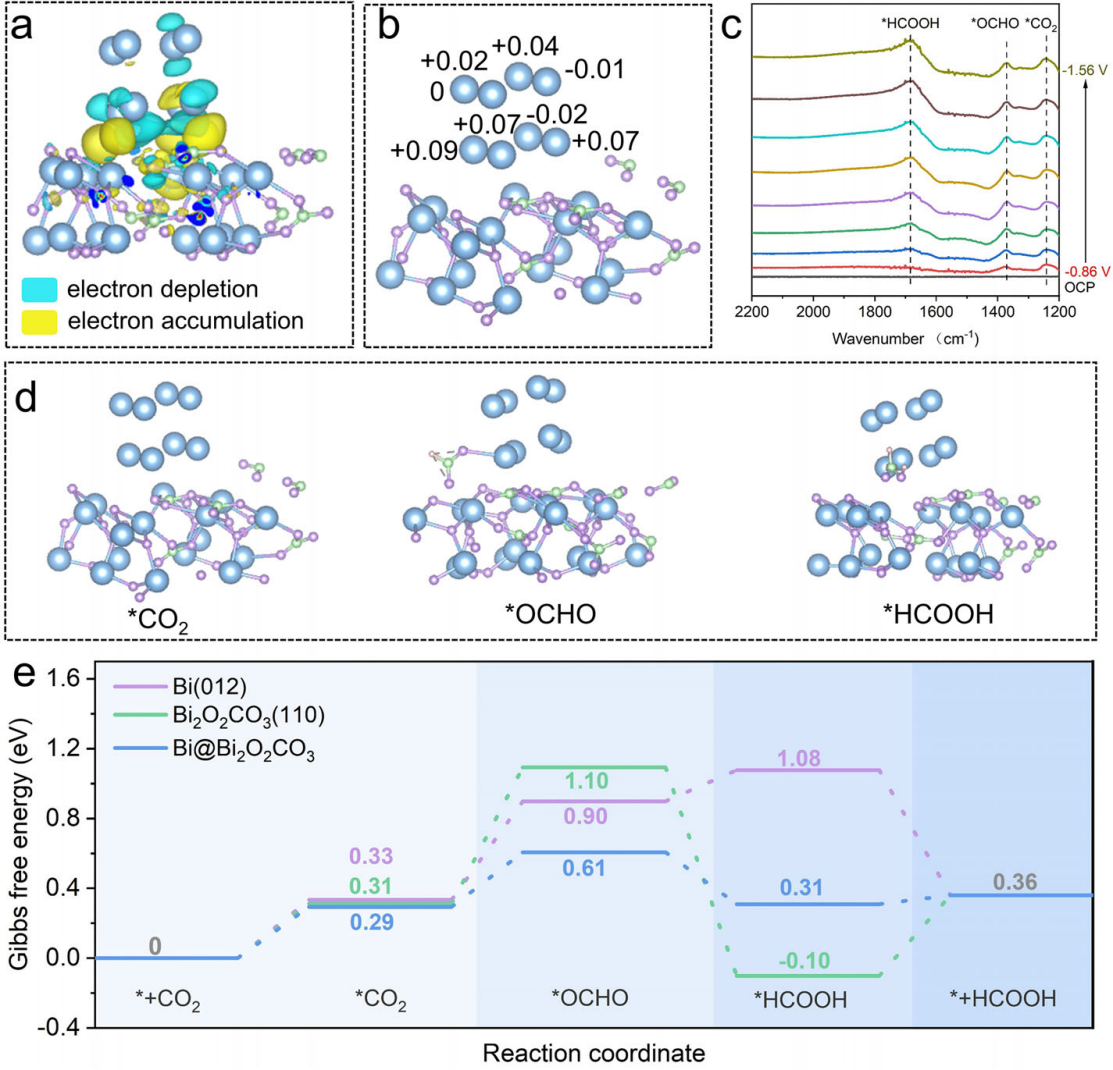
Mechanistic investigations for ECR to formate. a) Charge density difference of Bi@Bi_2_O_2_CO_3_. b) Bader charges of Bi atoms in Bi@Bi_2_O_2_CO_3_ (positive values represent electron loss). c) In situ FTIR spectra monitoring reaction intermediates. d) Optimized structure and adsorbed intermediates on Bi@Bi_2_O_2_CO_3_. e) Gibbs free energy diagram for ECR to formate on Bi (012), Bi_2_O_2_CO_3_(110), and Bi@Bi_2_O_2_CO_3_, respectively.

Density functional theory (DFT) calculations were employed to investigate the mechanisms of ECR to formate. Bi (012), Bi_2_O_2_CO_3_ (110), and Bi@Bi_2_O_2_CO_3_ were selected for DFT calculations (Figure S15, Supporting Information). Charge density difference analysis clearly showed regions of electron depletion (blue areas) on Bi and electron accumulation (yellow areas) on Bi_2_O_2_CO_3_, indicating that strong electron transfer occurred from Bi to Bi_2_O_2_CO_3_ (**Figure** **5**a). Bader charge analysis further quantified this process, revealing that Bi atoms lost ≈0.263 |e| (Figure 5b; Figure S16 and Table S2, Supporting Information), with the transferred electrons accumulating on the adjacent Bi atoms within the Bi_2_O_2_CO_3_ layer. This charge redistribution at the Bi@Bi_2_O_2_CO_3_ interface was consistent with the observed shift in Bi─O binding energy and is crucial for modulating intermediate adsorption and catalytic activity.

To elucidate the reaction pathway for the electrochemical conversion of CO_2_ to formate, in situ Fourier‐transform infrared (FTIR) spectroscopy was employed to identify key intermediates. As shown in Figure 5c, distinct infrared absorption bands were observed under applied potentials, with peaks centered at 1241, 1371, and 1685 cm^−1^ corresponding to ^*^CO_2_, ^*^OCHO, and ^*^HCOOH, respectively.^[^ ^58^, ^59^ ^]^ Thus, for the ECR to formate, CO_2_ first adsorbs onto the catalyst surface to form ^*^CO_2_, which is subsequently reduced to the ^*^OCHO intermediate through the electron and proton‐coupling process. This intermediate then hydrogenates to form ^*^HCOOH, followed by surface desorption to yield HCOOH, consistent with previous work.^[^ ^60^, ^61^, ^62^, ^63^, ^64^ ^]^

We optimized the structures of various reaction intermediates on Bi (012), Bi_2_O_2_CO_3_ (110), and Bi@Bi_2_O_2_CO_3_ surfaces (Figure 5d; Figure S17, Supporting Information), and calculated the Gibbs free energy change (ΔG) at each step of the ECR process, incorporating solvent effects^[^ ^65^, ^66^, ^67^ ^]^ (Figure 5e; Table S3, Supporting Information). The bond lengths between ^*^CO_2_ and the surface Bi atoms were comparable among the three surfaces (Figure S18, Supporting Information). Similarly, the ΔG values for ^*^CO_2_ formation differed only slightly (0.33, 0.31, and 0.29 eV, respectively), indicating that the interaction strengths with CO_2_ were essentially equivalent among these surfaces. The formation of the ^*^OCHO intermediate exhibited significant differences among the models. The energy barriers on Bi (012) and Bi_2_O_2_CO_3_ (110) surfaces were relatively high (0.57 and 0.79 eV, respectively), whereas that on Bi@Bi_2_O_2_CO_3_ was much lower (0.32 eV). This indicates that the Bi@Bi_2_O_2_CO_3_ interface can more effectively stabilize the ^*^OCHO key intermediate, resulting in superior formate performance. Additionally, the hydrogenation of ^*^OCHO to ^*^HCOOH on Bi@Bi_2_O_2_CO_3_ exhibited a favorable ΔG value of −0.30 eV, indicating that once ^*^OCHO formed, ^*^HCOOH generation proceeded spontaneously. The desorption of HCOOH from the surface was thermodynamically nearly barrierless, suggesting a facile desorption process. The adsorption energy of the key intermediates on the three surfaces was also calculated (Table S4, Supporting Information). The Bi@ Bi_2_O_2_CO_3_ interface exhibited the strongest adsorption for the ^*^OCHO intermediate (−2.69 eV), in contrast to Bi (−2.40 eV) and Bi_2_O_2_CO_3_ (−2.20 eV). Meanwhile, the desorption of ^*^HCOOH on Bi@Bi_2_O_2_CO_3_ (0.14 eV) was moderate and favorable for efficient product release. Briefly, the charge redistribution between Bi and Bi_2_O_2_CO_3_ alters the adsorption behavior of reaction intermediates, which facilitates the subsequent proton‐coupled electron transfer of ^*^CO_2_ and the desorption of ^*^HCOOH, thus exhibiting the excellent ECR performance of the catalyst for formate production.

### 2.5 Production of Electrolyte‐Free Formic Acid

**Figure 6:**
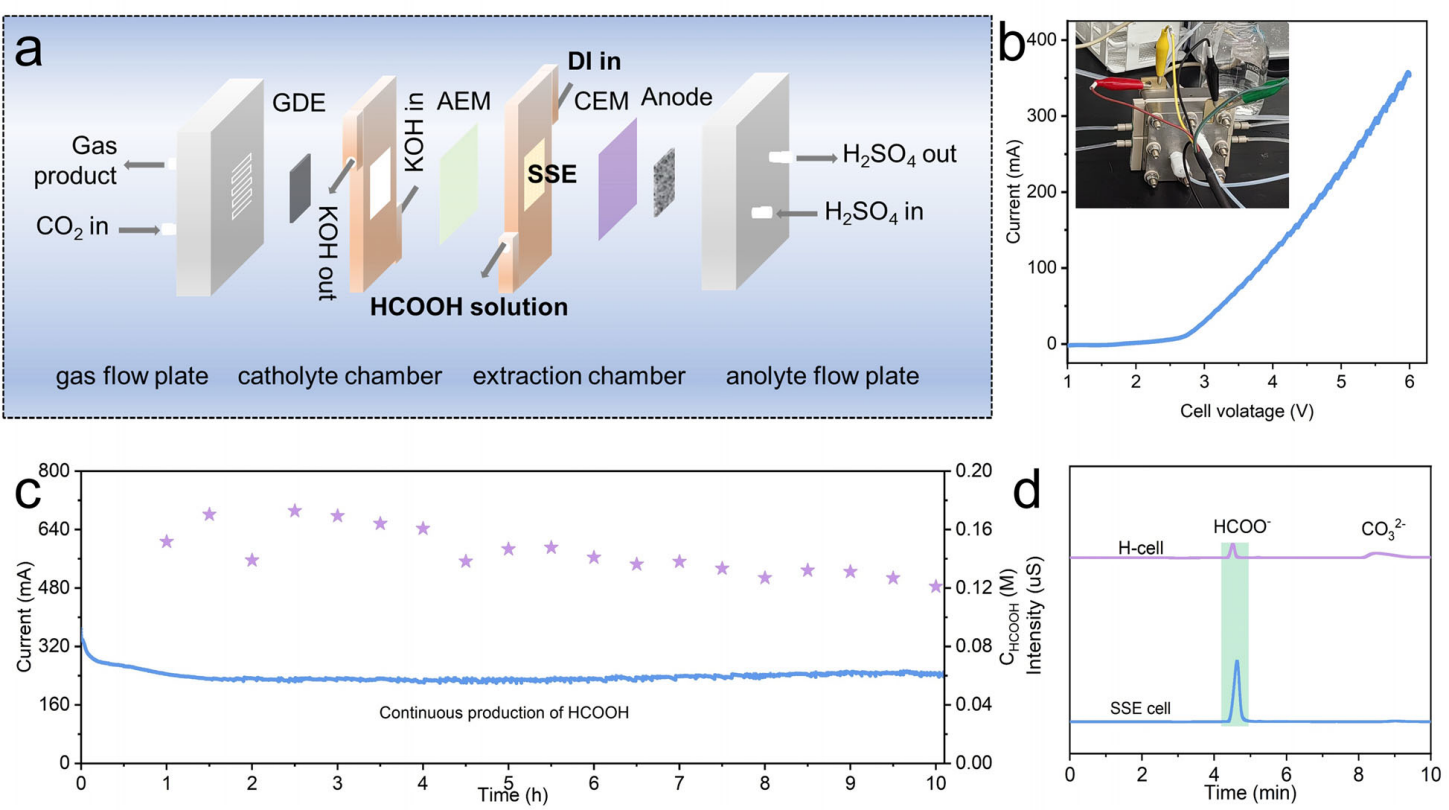
SSE cell for electrolyte‐free formic acid production. a) Schematic illustration of the customized SSE cell. b) LSV curve of the BOS_R_ catalyst (inset: photograph of the assembled system). c) Continuous electrolysis at a constant potential. d) Ion chromatography spectra of liquid products obtained from the H‐cell and SSE cell.

The BOS_R_ catalyst exhibits excellent selectivity and high catalytic activity toward formate production during ECR, highlighting the prospects for practical implementation. However, in conventional electrochemical systems, the liquid‐phase products inevitably coexist with electrolyte ions such as HCO_3_^−^ and K^+^, necessitating complex and energy‐intensive purification processes. To overcome this limitation, we developed an electrolytic cell integrated with solid‐state electrolytes (SSE cell), enabling direct production of electrolyte‐free formic acid (HCOOH). A schematic representation of the SSE cell configuration is shown in **Figure** **6**a.

The anode consisted of a commercially available 0.25 mm‐thick platinum–titanium felt coated with a 0.5 µm‐thick platinum layer, which serves as the site for proton (H^+^) generation via the oxygen evolution reaction. The cathode, coated with the BOS_R_ catalyst, facilitates the electroreduction of CO_2_ to HCOO^−^. Cost‐effective anion exchange membrane (AEM) and cation exchange membrane (CEM) materials (TWEDA and TWEDC) were employed to facilitate ion transport between the cathode and anode chambers. Under the applied electric field, HCOO‐ and H^+^ migrate through the AEM and CEM into the extraction chamber, which is filled with a porous solid‐state electrolyte (SSE). SSE is composed of commercially available spherical styrene‐divinylbenzene copolymers with sulfonic acid functional groups (Amberchorm 50WX8, hydrogen form), which facilitates ion conduction (Figure S19, Supporting Information). Within the 2 mm‐thick SSE layer, which offers both low internal resistance and structural integrity,^[^ ^68^ ^]^ HCOO^−^ and H^+^ recombine ionically to form HCOOH, which then diffuses with flowing deionized water and is collected.

Electrochemical performance of the SSE cell was evaluated by LSV, as shown in Figure 6b. The current increased continuously with rising cell voltage, exceeding 350 mA at 6 V. The relatively high operating voltage is mainly attributed to limited ion mobility within the SSE and the higher resistance of low‐cost ion exchange membranes.^[^ ^69^ ^]^ Despite this, the elimination of downstream separation steps offsets the increased energy input at the cell level, resulting in competitive overall energy efficiency. Subsequently, continuous electrolysis was performed at a constant voltage (Figure 6c). The current remained stable at ≈240 mA throughout the operation, showing no significant decay over time. The liquid product collected from the SSE cell outlet was analyzed by ion chromatography (IC) after appropriate dilution. A single distinct peak corresponding to formic acid was observed at ≈4.6 min, with no significant byproduct peaks detected. In contrast, the liquid product from a conventional H‐cell exhibited a strong carbonate peak at ≈8.5 min, as shown in Figure 6d, confirming that the product obtained from the SSE cell was of high‐purity formic acid. Quantitative analysis showed a steady HCOOH concentration above 0.12 m throughout the electrolysis. The measured pH was ≈2.30, which closely matches the theoretical value of 2.34 for a 0.12 m HCOOH solution, further confirming the high purity of the product. Overall, the integration of the BOS_R_ catalyst with an SSE cell enables the continuous and stable production of high‐purity, electrolyte‐free formic acid at appreciable concentrations. This system effectively offers promising prospects for industrial‐scale applications and product utilization.

## 3 Conclusion

In this work, layered bismuth silicate (Bi_2_SiO_5_) was designed as a pre‐catalyst for electrochemical reconstruction to form a Bi@Bi_2_O_2_CO_3_ composite, whose interface underwent charge redistribution to promote the proton‐coupled electron transfer of ^*^CO_2_ and promotes the desorption of ^*^HCOOH, thereby significantly improving the electrocatalytic performance of the catalyst for formate production. The catalyst can achieve a formate Faradaic efficiency of 95.8% at −1.06 V versus RHE while maintaining high selectivity within the wide potential window (−0.86 V to −1.26 V) in H‐cell and at high current density operation (50–300 mA cm^−2^) in the flow cell. Additionally, integrating the catalyst into an electrolytic cell with solid‐state electrolytes enables the continuous production of electrolyte‐free formic acid solution, thus avoiding energy‐consuming purification processes. This study not only reveals the reconstruction mechanism of layered bismuth silicate but also provides a scalable strategy to combine efficient electrocatalysis with sustainable product recovery for practical carbon utilization. Developing solid electrolytes with more conductive solid‐electrolyte particles will be essential for reducing ohmic losses and further improving the energy efficiency of CO_2_ electrolysis technologies.

## Conflict of Interest

The authors declare no conflict of interest.

## Supporting information



Supporting Information

## Data Availability

The data that support the findings of this study are available in the supplementary material of this article.
