# Supplementary material for: Bismuth Silicate Catalyst for Efficient Electrocatalytic CO2 Reduction and Electrolyte‐Free Formic Acid Production
Source: Adv Sci (Weinh). 2025 Aug 11;12(41):e06034. doi: 10.1002/advs.202506034 (PMC12591196; doi:10.1002/advs.202506034)
Supplement: Supplementary file 1 — Supporting Information [file ADVS-12-e06034-s001.docx]

**Supporting information**

**Bismuth Silicate Catalyst for** **Efficient Electrocatalytic CO_2_ Reduction and Electrolyte-Free Formic Acid Production**

*Ping Zhu^a^, Xin-Hao Cai^a^, Cheng-Cheng Huang^a^, Ying Zhou^b^, Na Chu^c^, Zi-Bo Jing^a^, Wen-Long Wang^a^, Bilu Liu^d^,Yong Jiang^e^*, Qian-Yuan Wu^a^**

^a^ Shenzhen Key Laboratory of Ecological Remediation and Carbon Sequestration, Key Laboratory of Microorganism Application and Risk Control, Ministry of Ecology and Environment, State Key Laboratory of Regional Environment and Sustainability, Key Laboratory of Microorganism Application and Risk Control of Shenzhen, Guangdong Provincial Engineering Research Center for Urban Water Recycling and Environmental Safety, Institute of Environment and Ecology, Shenzhen International Graduate School, Tsinghua University, Shenzhen 518055, PR China

^b^ Department of Materials Science and Technology, University of Science and Technology of China, Anhui 230026, PR China

^c^ Fujian Key Laboratory of Pollution Control and Resource Reuse, College of Environmental and Resource Sciences, Fujian Normal University, Fuzhou 350117, PR China

^d^ Institute of Materials Research, Tsinghua Shenzhen International Graduate School, Tsinghua University, Shenzhen 518055, PR China.

^e^ College of Resources and Environment, Fujian Agriculture and Forestry University, Fuzhou 350002, PR China.

*Corresponding author:

Yong Jiang, [jiangyongchange@fafu.edu.cn](mailto:jiangyongchange@fafu.edu.cn);

Qian-Yuan Wu, [wu.qianyuan@sz.tsinghua.edu.cn](mailto:wu.qianyuan@sz.tsinghua.edu.cn).

# Experimental Section

**Chemicals**

Bismuth nitrate pentahydrate (Bi(NO_3_)_3_·5H_2_O), sodium metasilicate nonahydrate (Na_2_SiO_3_.9H_2_O), potassium bicarbonate (KHCO_3_), sodium hydroxide (NaOH), potassium hydroxide (KOH) and ethylene glycol (C_2_H_6_O_2_) were obtained from Macklin Industrial Corporation. Porous styrenedivinylbenzene sulfonated copolymer microspheres (AmberchromTM 50WX8, hydrogen form) were purchased from Sigma-Aldrich. All the chemicals in these experiments were used without further purification. High purity carbon dioxide (CO_2_, 99.999%), nitrogen (N_2_, 99.999%) were supplied by Shenzhen Huatepeng Special Gas Co., Ltd., The water used in all experiments with a resistivity of 18.2 MΩ obtained from Mill-Q system.

**Catalyst preparation**

The catalyst was synthesized using a simple solvothermal method. Initially, Bi(NO_3_)_3_·5H_2_O (3 mmol) was dissolved in ethylene glycol (30 mL) and magnetically stirred for 30 minutes to form solution A. Meanwhile, Na_2_SiO_3_·9H_2_O (1.5 mmol) was dissolved in deionized water (30 mL) and magnetically stirred for 30 min to form solution B. Solution B was then slowly added dropwise to solution A under continuous stirring. The pH of the mixture was adjusted using NaOH solution (1 M) while maintaining stirring. The resulting mixture was transferred into a stainless-steel autoclave lined with polytetrafluoroethylene and heated at 200°C for 10 hours in an explosion-proof oven. After the reaction, the reactor was allowed to cool to room temperature naturally. The resulting product was then filtered, washed three times with absolute ethanol and deionized water, and dried overnight at 60°C. The obtained Bi_2_SiO_5_ were then immobilized onto the commercially available carbon paper (Sigracet 39BB) under an infrared lamp with a loading density of 1.0 mg cm^−2^ and subject to −1.06 V versus RHE for 1 h in CO_2_-saturated 0.5 M KHCO_3_ solution to electrochemically transform the loaded Bi_2_SiO_5_ into BOS_R_. The catalyst ink was prepared by dispersing 5mg powder in a mixture of 950 μL isopropanol and 50 μL 5% Nafion solution.

**Material characterizations**

The morphology of the materials was characterized using scanning electron microscope coupled with energy dispersion spectrometer (SEM, Zeiss SUPRA 55), high-resolution transmission electron microscope (HRTEM, Talos F200S) and High-angle annular dark-field scanning transmission electron microscopy (HAADF-STEM, JEM-ARM200F). The structural properties were analyzed using powder X-ray diffraction (XRD, SmartLab), X-ray photoelectron spectroscopy (XPS, Thermo Scientific K-ALPHA, Al Kα excitation source) and Fourier transform infrared spectrometer (FT-IR, NICOLET iS50).

**Electrochemical** **measurement**

In the H-cell, a platinum sheet (2 cm × 2 cm) and Ag/AgCl (3.5 M KCl) electrode were used as the counter electrode and reference electrode, respectively. Each cell contained 20 mL of 0.5 M KHCO_3_ solution, separated by a proton exchange membrane (Nafion N117) between the anolyte and catholyte. Prior to each experiment, the system was checked for airtightness, and the KHCO_3_ solution was saturated by bubbling high-purity CO_2_ at a flow rate of 100 mL min^−1^ for at least 30 minutes. During the test, the flow rate of CO_2_ was maintained at 20 mL min^−1^. Unless otherwise specified, all potentials reported in this study have been converted to reversible hydrogen electrodes (RHE) without IR compensation. The electrochemical active surface area (ECSA) was estimated by measuring the double-layer capacitance (C_dl_) at 0–0.1 V versus Ag/AgCl with the scan rates ranging from 20 to 120 mV s^-1^.

In the flow cell, a platinum sheet (2 cm × 2 cm) and Hg/HgO electrode were used as the counter electrode and reference electrode, respectively. with 1 M KOH as the electrolyte. In the electrolytic cell containing solid-state electrolyte, a commercially available 0.25 mm thick platinum-titanium felt, coated with a 0.5 μm thick platinum layer, was used as the anode electrode. The catholyte and anolyte consisted of 1 M KOH and 0.5 M H_2_SO_4_, respectively. Ion exchange membranes (TWEDC and TWEDA) were utilized to facilitate ion transfer between the cathode and anode chambers. During the experiment, the flow rate of high-purity CO_2_ gas was controlled at 20 mL min^−1^. Low-cost anion exchange membrane (AEM) and cation exchange membrane (CEM) materials (TWEDA and TWEDC, sourced from a local supplier) were employed to facilitate ion transport between the cathode and anode chambers.

**Product analysis**

A customized gas chromatography system (GC2014, Shimadzu) was employed for online detection of gaseous products during the electrochemical CO_2_ reduction process. This system is equipped with a ten-port valve, three chromatographic columns, a methane converter (MTN), two flame ionization detectors (FID), and a thermal conductivity detector (TCD). It enables the detection of common CO_2_ reduction products, including H_2_, CH_4_, CO, C_2_H_4_, and C_2_H_6_. The liquid product was qualitatively determined by proton nuclear magnetic resonance spectroscopy (Bruker AVANCE NEO 400MHz). The quantitative detection of formic acid was conducted using ion chromatography (IC) on a Thermo Scientific Dionex Aquion RFIC system. The separation of analytes was achieved using a Dionex IonPac AS15 analytical column (4 mm × 250 mm) coupled with a Dionex IonPac AG19 guard column (4 mm × 50 mm), both supplied by Thermo Scientific. An AERS 400 anion electrolytic suppressor was used, with the suppression current set at 38 mA. A high-purity KOH eluent (0.015 M) was generated using an eluent generator, and the flow rate was maintained at 1.0 mL min^-1^.

The Faradaic efficiencies (FE) for the liquid product were determined using the equation:

$$\text{FE}_{\text{formate}}\text{=}\frac{\text{2×F×}\text{c}\text{×V}}{\text{Q}}\text{×100\%}$$

where F is the Faraday’s constant (96485 C/mol), c is the concentration of formate, V is the volume of the electrolyte, and Q is the total charge involved in the CO_2_ electroreduction.

***In situ* FTIR measurement**

Electrochemical *in situ* FTIR spectroscopy measurements were performed on a Nicolet iS50 Fourier-transform infrared spectrometer equipped with a mercury-cadmium-telluride (MCT/A) detector cooled with liquid N_2_ and a VeeMax III (PIKE Technologies) accessory. The sample to be tested was sealed with a conductive tape and placed under carbon paper coated with catalyst as working electrode, which was vertically pressed on the CaF_2_ window plate. A platinum mesh and an Ag/AgCl electrode were used as the counter electrode and reference electrode, respectively. Before testing, the detector was cooled with liquid nitrogen for at least 30 min to maintain a stable signal. The incoming infrared beam was approximately aligned with the normal electrode surface. Chronoamperometry was used for CO_2_RR test and was accompanied by the spectrum collection (32 scans, 4 cm^-1^ resolution). All spectra were subtracted with the background. Spectral recording was performed on a Thermo Scientific Nicolet iS50 spectrometer.

**Theoretical calculation methods**

All calculations were performed using density functional theory (DFT) in conjunction with the projector augmented plane-wave (PAW) method^[1]^. Models of (2×3) double-layer Bi_2_O_2_CO_3_(110) slab, (3×1) three-layer Bi (012) slab, and Bi_2_O_2_CO_3_(110) slab were built. To prevent artificial interactions between periodic images, a vacuum layer of 20 Å was added perpendicular to the sheet. The plane wave cut-off energy and energy criterion were set to 400 eV and 10^-5^ eV, respectively. Brillouin zone integration was performed using a 2 × 2 × 1 K-mesh. All structures were fully relaxed until the residual forces on the atoms were reduced to less than 0.02 eV Å−1. For the calculation of Gibbs free energy differences (ΔG) associated with the elementary reaction steps, we utilized the computational hydrogen electrode model established by Nørskov et al^[2]^. The Poisson-Boltzmann implicit solvation model, Vaspsol^[3]^, was employed to investigate the effect of solvation as implemented in VASP, where the dielectric constant was set to 5.6 for KHCO_3_.

# Supplementary Figures


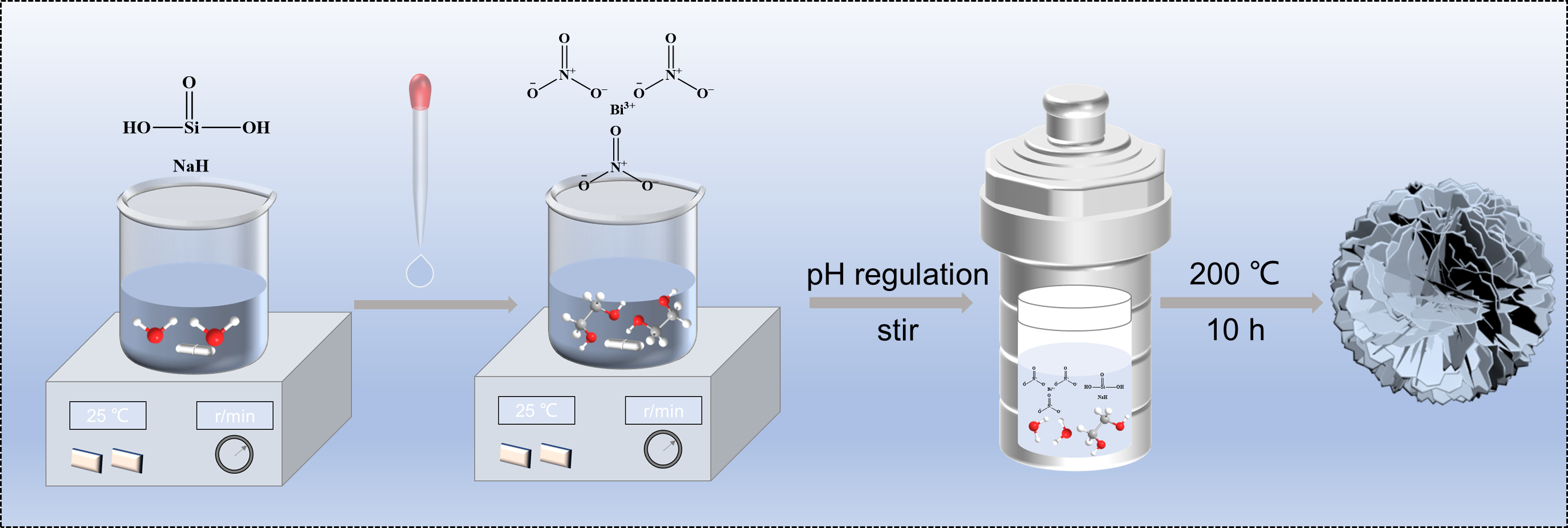


Figure S1. Schematic of the synthesis process of bismuth silicate (Bi_2_SiO_5_).


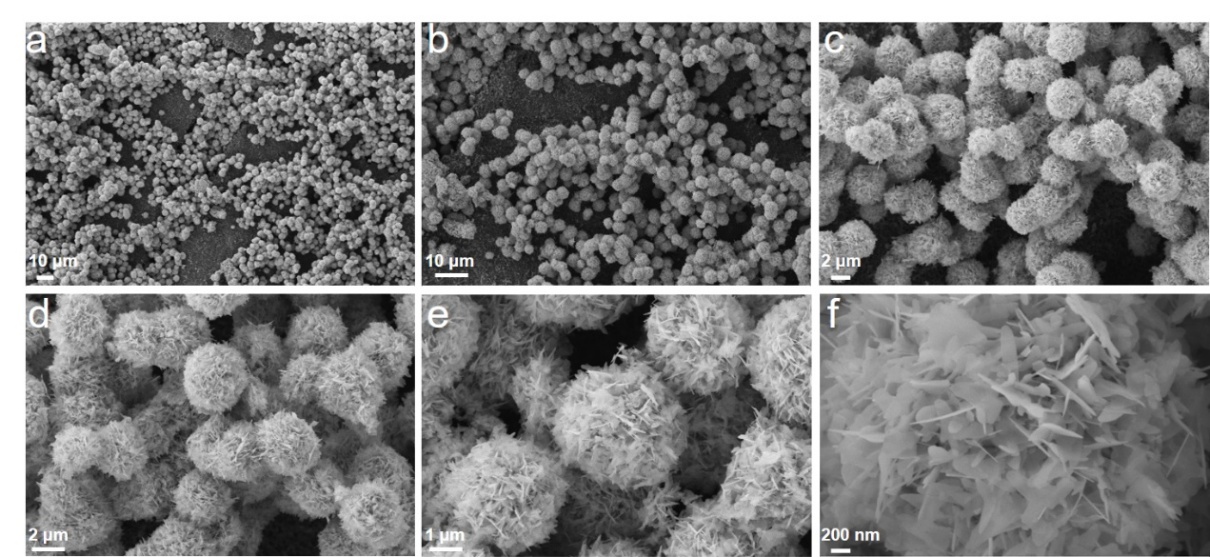


Figure S2. SEM images of Bi_2_SiO_5_ at different magnification.


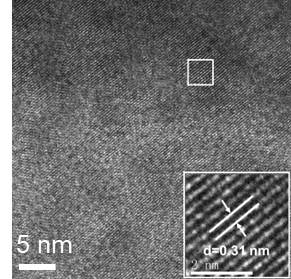


Figure S3. HRTEM image of the Bi_2_SiO_5_.


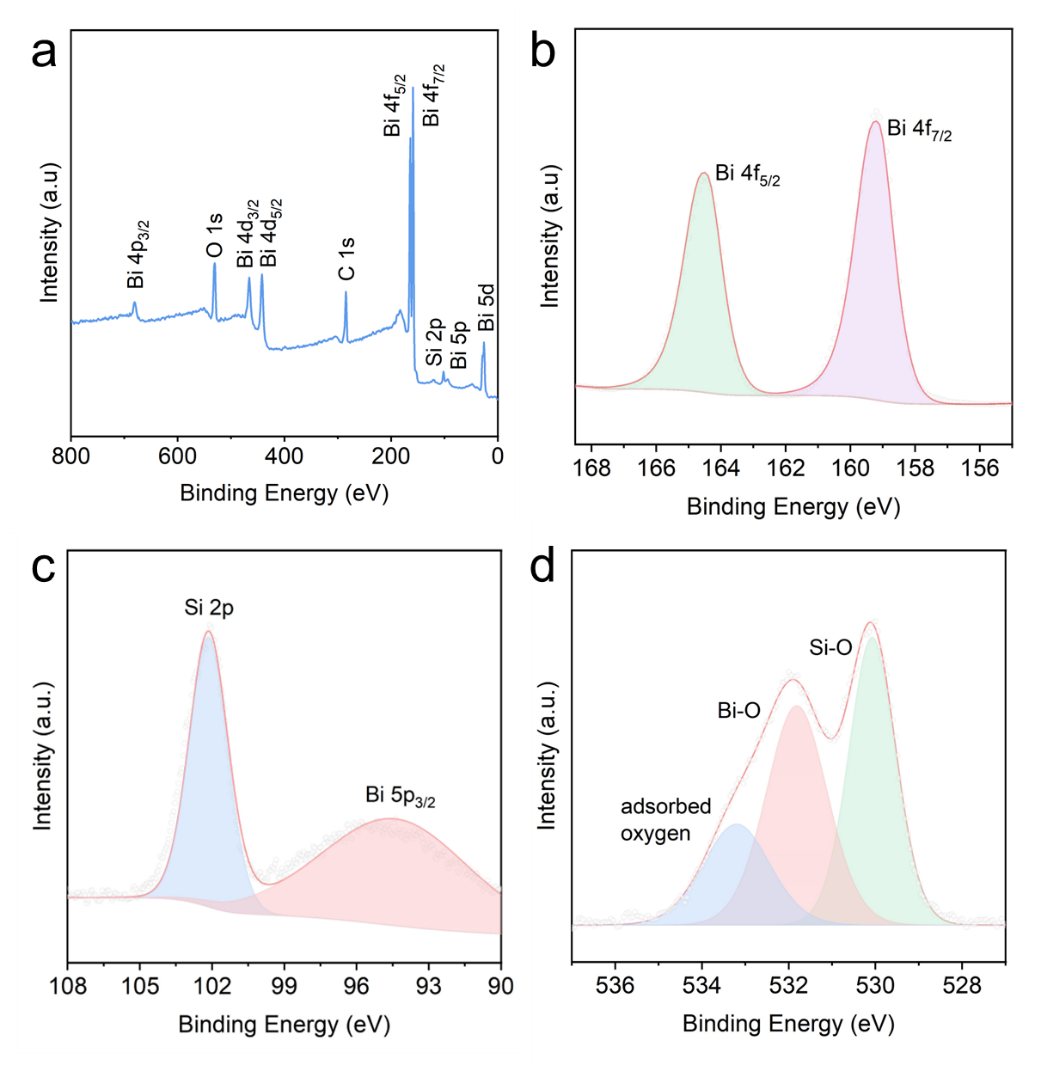


Figure S4. XPS survey spectra of the Bi_2_SiO_5_. a) survey spectrum, b) Bi 4f, c) Si 2p, d) O 1s.


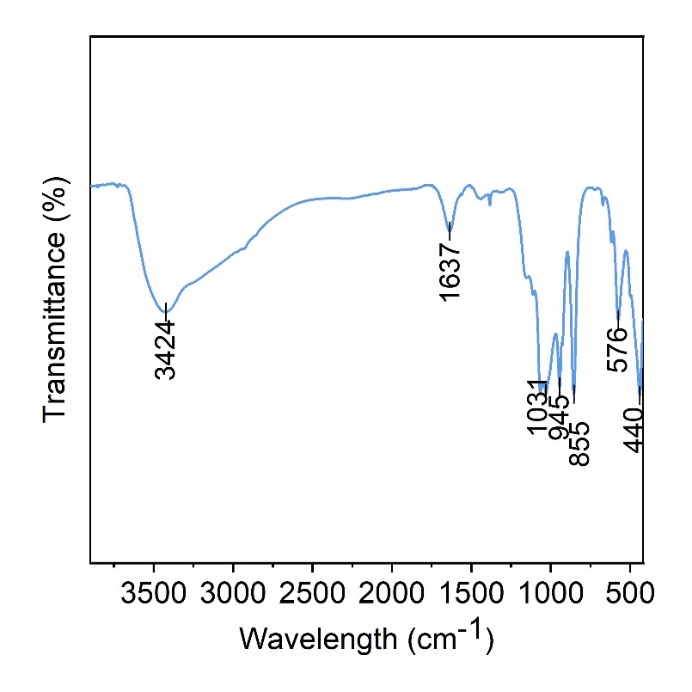


Figure S5. FTIR spectra of the Bi_2_SiO_5_. The broad absorption band around 3424 cm^-1^ is caused by the stretching and bending vibrations of the O-H bond caused by the oxygen-containing species adsorbed on the surface. The peak at 1637 cm^-1^ is the vibration peak of water molecules, and the peaks around 440 cm^-1^, 576 cm^-1^, 855 cm^-1^, 945 cm^-1^, and 1031 cm^-1^ represent the stretching vibration modes of the Bi−O bond, (SiO_4_)^4-^ group, Bi−O−Si bond, (SiO_5_)^6−^ group, and Si−O bond, respectively.


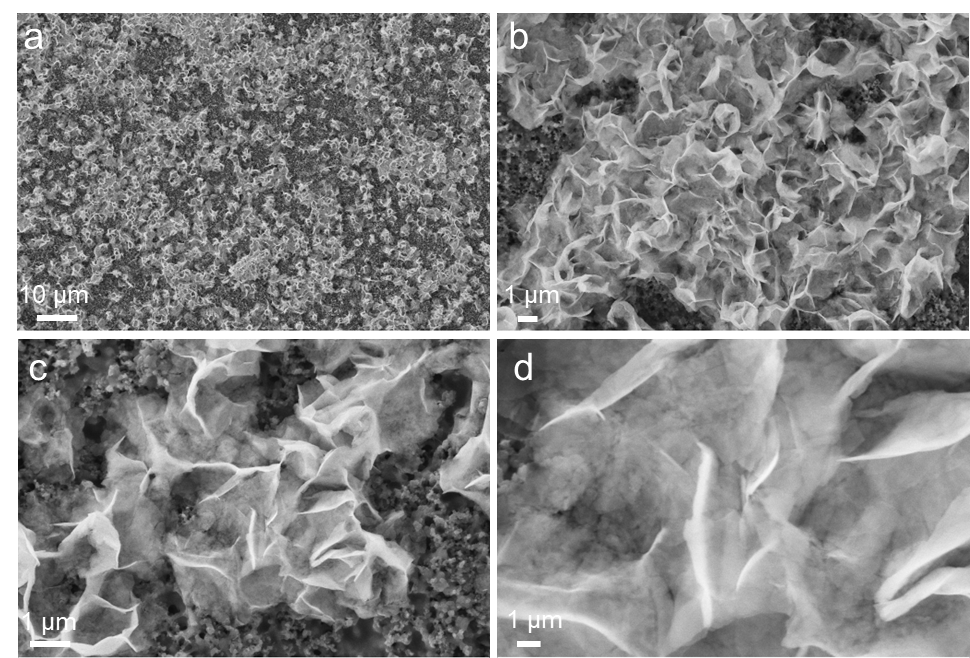


Figure S6. SEM images of the reconstructed catalyst (BOS_R_) at different magnifications.


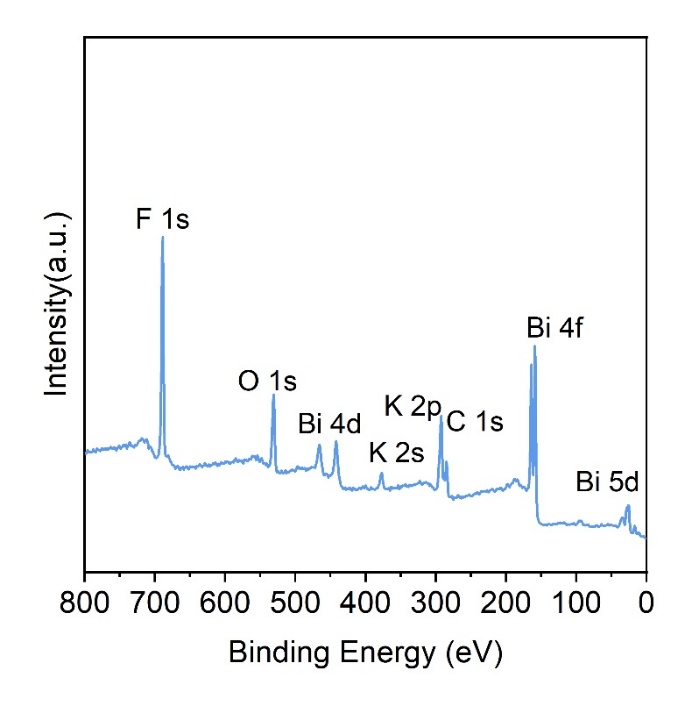


Figure S7. The XPS survey spectrum of the BOS_R_ catalyst.


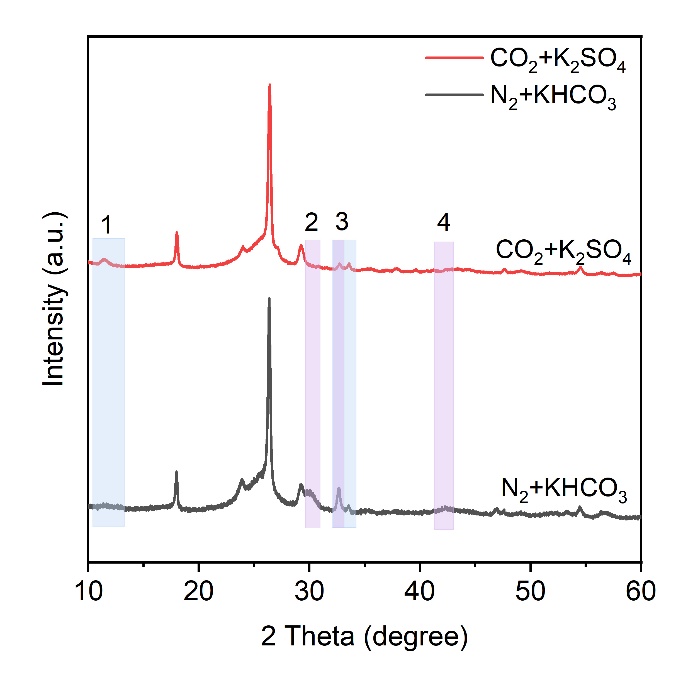


Figure S8. XRD patterns of the Bi_2_SiO_5_ electrode after electrolysis under various conditions. Characteristic peaks of Bi_2_SiO_5_ and Bi_2_O_2_CO_3_ are marked in blue and purple, respectively.


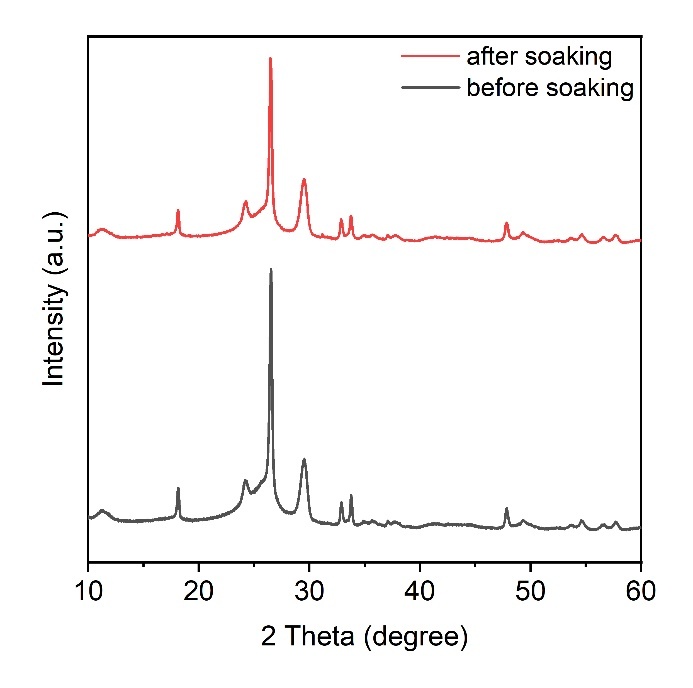


Figure S9. XRD patterns for Bi_2_SiO_5_ before and after soaking in 0.5 M KHCO_3_ electrolyte.


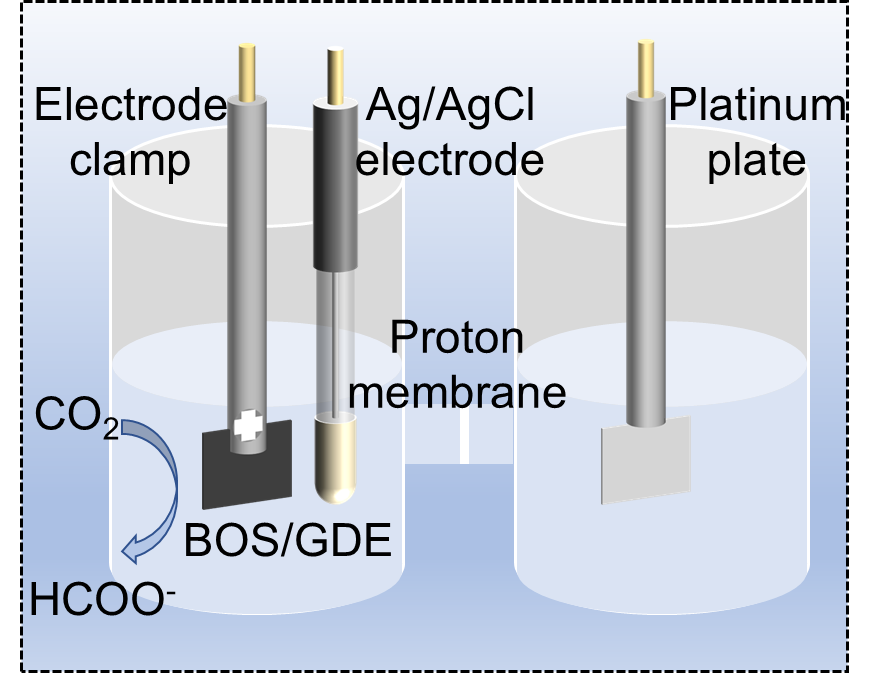


Figure S10. Schematic illustration of the H-cell configuration.


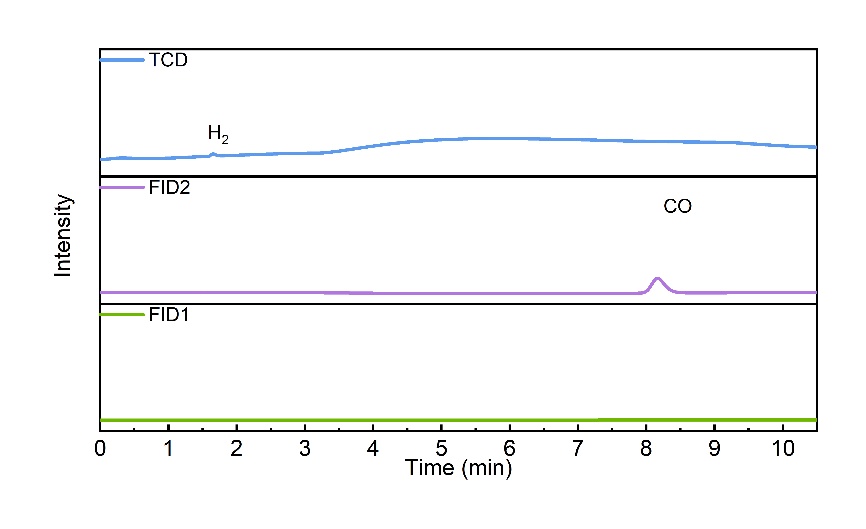


Figure S11. Analysis of gas-phase products of the BOS_R_ catalyst by GC.


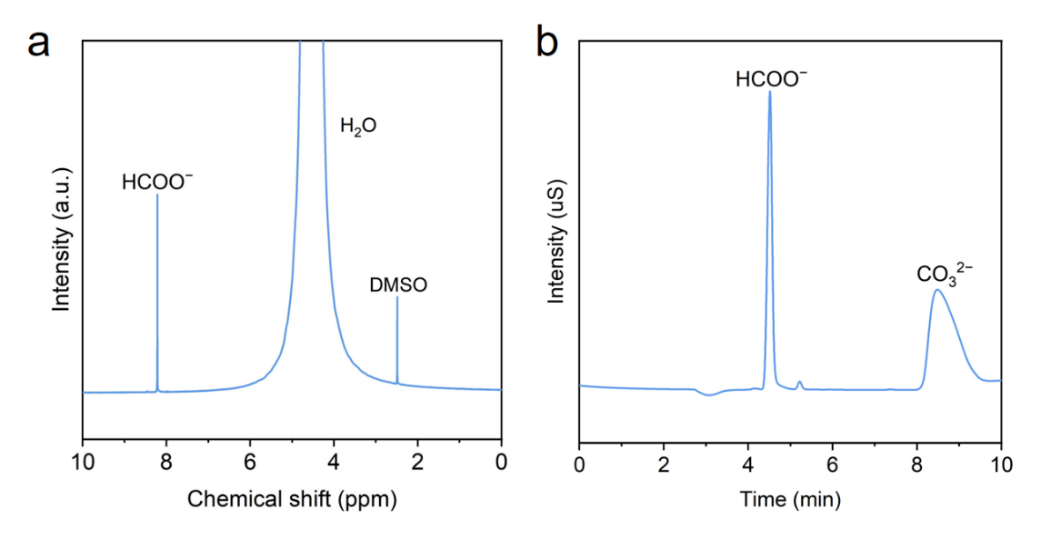


Figure S12. Analysis report of liquid-phase products of the BOS_R_ catalyst by ^1^H NMR.


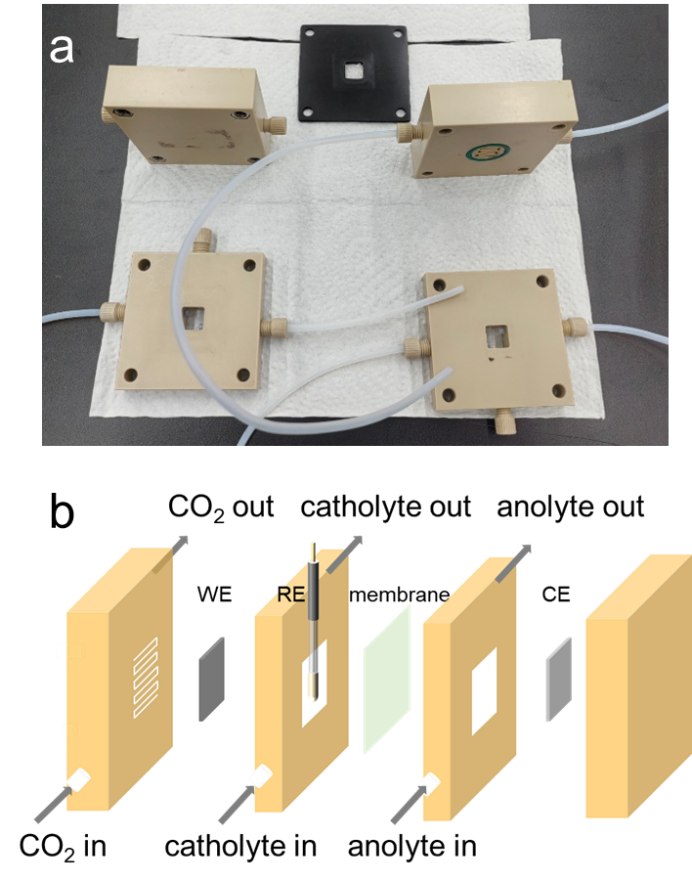


Figure S13. a) Optical image of the assembled flow cell. b) Schematic illustration of the flow cell configuration.


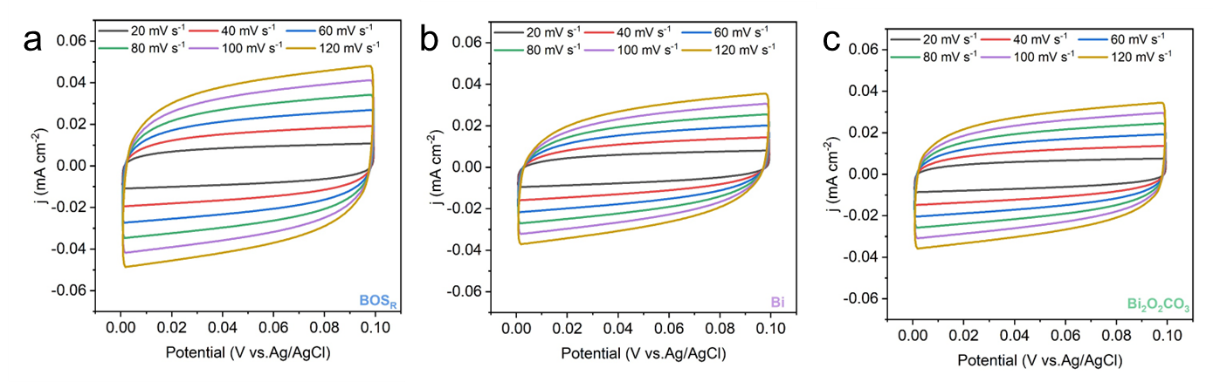


Figure S14. Cyclic voltammetry curves for a) BOS_R_, b) Bi, c) Bi_2_O_2_CO_3_ measured at 0–0.1 V versus Ag/AgCl with the scan rates ranging from 20 to 120 mV s^-1^.


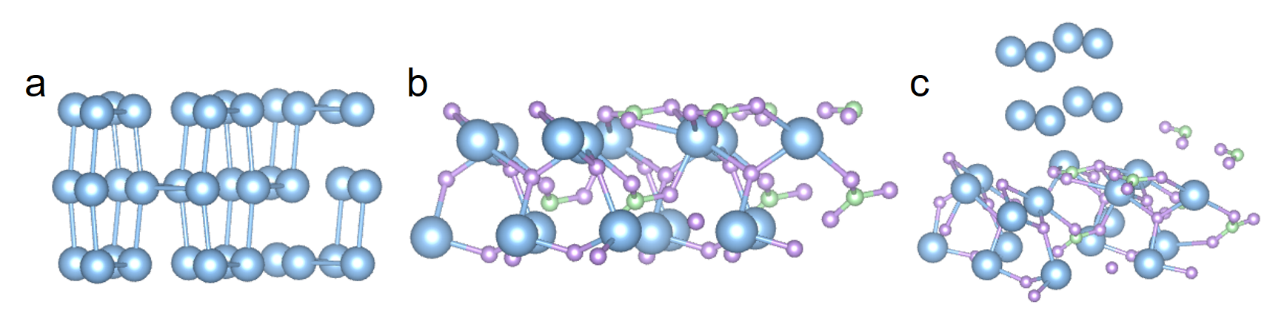


Figure S15. The optimized crystal structure of a) Bi (012), b) Bi_2_O_2_CO_3_ (110), c) Bi@ Bi_2_O_2_CO_3_ (110).


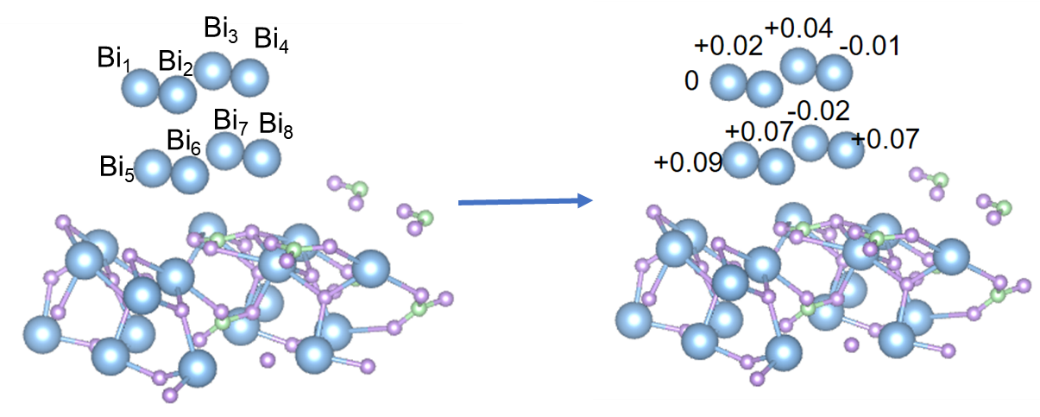


Figure S16. Bader charges corresponding to different Bi atoms in Bi@Bi_2_O_2_CO_3_.


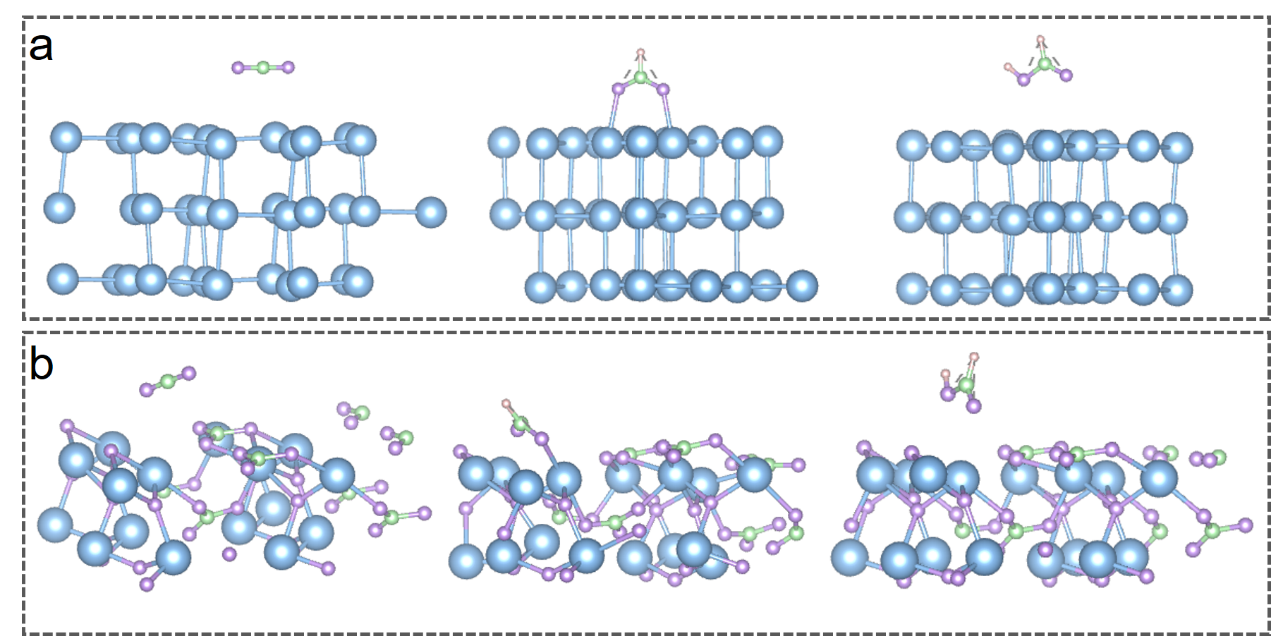


Figure S17. The optimized structure and the adsorbed intermediates on a) Bi (012), b) Bi_2_O_2_CO_3_ (110).


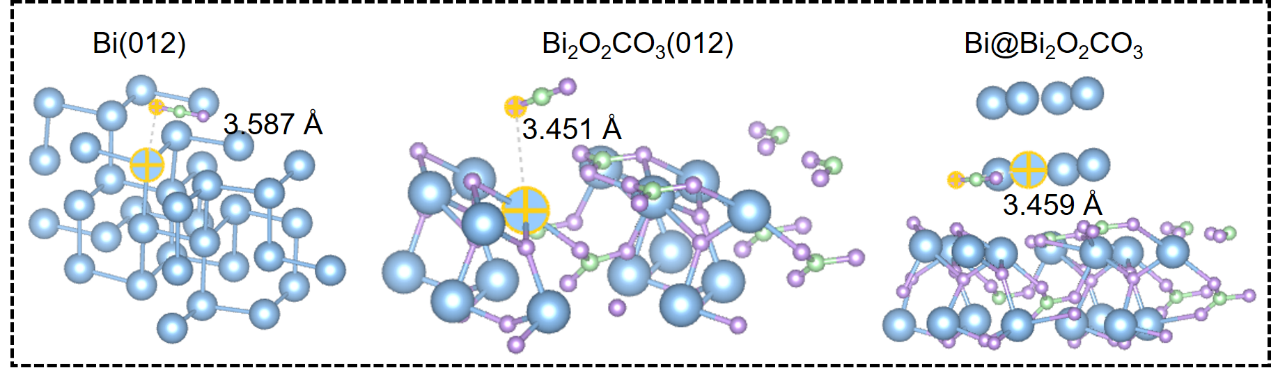


Figure S18.The bond length between *CO_2_ and the surface Bi atoms on Bi (012), Bi_2_O_2_CO_3_ (110) and Bi@ Bi_2_O_2_CO_3_.


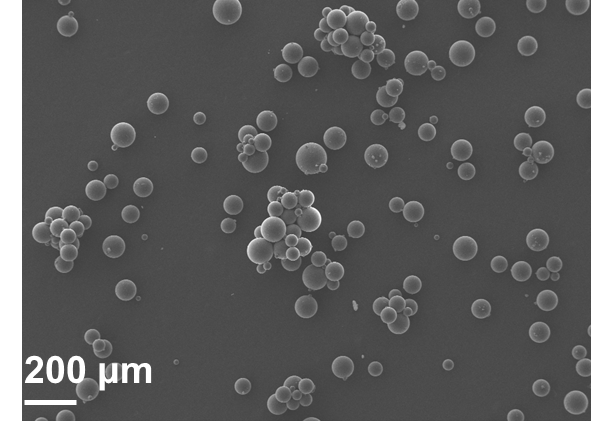


Figure S19. SEM image of solid-state electrolyte (Amberchorm@50 WX8, hydrogen form).

# Supplementary Tables

Table S1. Summary of reported Bi-based catalysts for ECR to formate in H-cell.

| Catalyst | Maximum FE_formate_ | Potential Range for FE_formate_ >90% (mV) | Ref |
| --- | --- | --- | --- |
| RD-Bi | 93.5% | 100 | ^[4]^ |
| PD-Bi_1_ | 91.4% | 100 | ^[5]^ |
| Bi_3_ | 96.47% | 300 | ^[6]^ |
| Bi_14_ | 92.76% | 200 | ^[6]^ |
| Bi-NFs | 92.3% | 200 | ^[7]^ |
| 3.5 nm Bi NS | 92% | 220 | ^[8]^ |
| 11 nm Bi NS | 87% | - | ^[8]^ |
| Bi@C-700-4 | 93.2% | 300 | ^[9]^ |
| Bi-NBs | 93.3% | 100 | ^[10]^ |
| Bi-NFs | 81.2% | - | ^[10]^ |
| Bi-MPs | 59.2% | - | ^[10]^ |
| Bi-NRs@NCNTs | 90.9% | - | ^[11]^ |
| Bi granules | 88.5% | - | ^[11]^ |
| Bi(btb) | 95% | 200 | ^[12]^ |
| Bi-TiO_2_-700 | 95.6% | 400 | ^[13]^ |
| Bi/ATO | 95% | 100 | ^[14]^ |
| SOR Bi@C NPs | 95% | 440 | ^[15]^ |
| Bi dendrite | 89.0% | - | ^[16]^ |
| NTD-Bi | 93% | 300 | ^[17]^ |
| Bi-PVP/CC600 | 86% | - | ^[18]^ |
| Bi-ene-NW | 95% | 420 | ^[19]^ |
| BiNN-CFs | 92% | 100 | ^[20]^ |
| Bi_2_O_2_CO_3_ NS | 92.6% | 300 | ^[21]^ |
| BOC@GDY | 95.5% | 150 | ^[22]^ |
| BOC | 85.9% | - | ^[22]^ |
| bulk-BOC | 68.3% | - | ^[22]^ |
| BOCNS | 85% | - | ^[23]^ |
| BOC with V_O_ | 94% | 300 | ^[24]^ |
| BOB | 96% | 200 | ^[25]^ |
| BOI | 85% | - | ^[25]^ |
| BOC | 81% | - | ^[25]^ |
| BiO_x_/C | 95.9% | 330 | ^[26]^ |
| fractal-Bi_2_O_3_ | 87% | - | ^[27]^ |
| Bi_2_O_3_@C-800 | 92% | 100 | ^[28]^ |
| Bi_2_O_3_@PPy-2 NSs | 95.8% | 200 | ^[29]^ |
| Bi_2_O_3_NSs@MCCM | 93.8% | 300 | ^[30]^ |
| Bi/Bi_2_O_3_-CP | 90.4 % | 100 | ^[31]^ |
| Bi_2_O_3_ | 91% | - | ^[32]^ |
| Bi_2_S_3_–PPy | 95.18% | - | ^[33]^ |
| Bi_2_S_3_ derived Bi | 84% | - | ^[34]^ |
| Bi-MOF | 92.2% | - | ^[35]^ |
| Bi_5_O_7_I | 96.14% | 250 | ^[36]^ |
| Bi_4_I_16_ | 80% | - | ^[37]^ |
| Te-Bi NRs | 93.0 % | 300 | ^[38]^ |
| Bi_2_Te_3_ NRs | 77.5% | - | ^[38]^ |
| Bi_2_SiO_5_ | 95.8% | 400 | This work |

Table S2. Bader charges of Bi atoms in Bi@Bi_2_O_2_CO_3_.

| Atom | X | Y | Z | Charge | Valence electrons | Bader | Explanation |
| --- | --- | --- | --- | --- | --- | --- | --- |
| Bi_1_ | 4.284 | 9.147 | 10.424 | 14.997 | 15 | 0.003 | Bi_1_ lost 0.003 e |
| Bi_2_ | 4.312 | 6.079 | 10.706 | 14.978 | 15 | 0.022 | Bi_2_ lost 0.022 e |
| Bi_3_ | 7.342 | 9.209 | 10.721 | 14.963 | 15 | 0.037 | Bi_3_ lost 0.037 e |
| Bi_4_ | 7.373 | 6.143 | 10.996 | 15.011 | 15 | -0.011 | Bi_4_ gain 0.011e |
| Bi_5_ | 4.587 | 8.879 | 7.363 | 14.911 | 15 | 0.089 | Bi_5_ lost 0.089 e |
| Bi_6_ | 4.618 | 5.812 | 7.644 | 14.926 | 15 | 0.074 | Bi_6_ lost 0.074 e |
| Bi_7_ | 7.639 | 8.920 | 7.677 | 15.019 | 15 | -0.019 | Bi_7_ gain 0.019 e |
| Bi_8_ | 7.673 | 5.866 | 7.940 | 14.930 | 15 | 0.070 | Bi_8_ lost 0.070 e |

Table S3. The Gibbs free energy change (ΔG) for the steps of ECR at the Bi (012), Bi_2_O_2_CO_3_ (110) and Bi@ Bi_2_O_2_CO_3_ (Unit: eV).

| Steps | Bi(012) | Bi_2_O_2_CO_3_ (110) | Bi@ Bi_2_O_2_CO_3_ |
| --- | --- | --- | --- |
| CO_2_+*→*CO_2_ | 0.33 | 0.31 | 0.29 |
| *CO_2_+H^+^+e^-^→*OCHO | 0.57 | 0.79 | 0.32 |
| *OCHO+H^+^+e^-^→*HCOOH | 0.18 | − 1.20 | − 0.30 |
| *HCOOH→*+HCOOH | − 0.72 | 0.46 | 0.05 |

Table S4. The adsorption energy (*E*_ads_) of key intermediates on Bi (012), Bi_2_O_2_CO_3_(110), and Bi@Bi_2_O_2_CO_3_ (Unit: eV).

| Intermediates | Bi(012) | Bi_2_O_2_CO_3_ (110) | Bi@Bi_2_O_2_CO_3_ |
| --- | --- | --- | --- |
| *CO_2_ | 0.67 | 0.65 | 0.63 |
| *OCHO | -2.40 | -2.20 | -2.69 |
| *HCOOH | 0.61 | -0.56 | 0.14 |

**References**

[1] G. Kresse, D. Joubert, *Phys. Rev. B* **1999**, 59, 1758.

[2] J. K. Nørskov, J. Rossmeisl, A. Logadottir, L. Lindqvist, J. R. Kitchin, T. Bligaard, H. Jonsson, *J. Phys. Chem. B* **2004**, 108, 17886.

[3] a) K. Mathew, R. Sundararaman, K. Letchworth-Weaver, T. Arias, R. G. Hennig, *J. Chem. Phys.* **2014**, 140, 084106; b) W. Guo, S. Zhang, J. Zhang, H. Wu, Y. Ma, Y. Song, L. Cheng, L. Chang, G. Li, Y. Liu, *Nat. Commun.* **2023**, 14, 7383; c) S. Yang, Z. Liu, H. An, S. Arnouts, J. De Ruiter, F. Rollier, S. Bals, T. Altantzis, M. C. Figueiredo, I. A. Filot, *ACS Catal.* **2022**, 12, 15146.

[4] C. Zhang, X. Hao, J. Wang, X. Ding, Y. Zhong, Y. Jiang, M. C. Wu, R. Long, W. Gong, C. Liang, *Angew. Chem. Int. Ed.* **2024**, 63, e202317628.

[5] Y. Wang, Y. Li, J. Liu, C. Dong, C. Xiao, L. Cheng, H. Jiang, H. Jiang, C. Li, *Angew. Chem. Int. Ed.* **2021**, 60, 7681.

[6] J.-W. Shi, S.-N. Sun, J. Liu, Q. Niu, L.-Z. Dong, Q. Huang, J.-J. Liu, R. Wang, Z. Xin, D. Zhang, *ACS Catal.* **2022**, 12, 14436.

[7] S. Yang, M. Jiang, W. Zhang, Y. Hu, J. Liang, Y. Wang, Z. Tie, Z. Jin, *Adv. Funct. Mater.* **2023**, 33, 2301984.

[8] D. Yao, C. Tang, A. Vasileff, X. Zhi, Y. Jiao, S. Z. Qiao, *Angew. Chem. Int. Ed.* **2021**, 60, 18178.

[9] W. Guo, X. Cao, D. Tan, B. Wulan, J. Ma, J. Zhang, *Angew. Chem. Int. Ed.* **2024**, 63, e202401333.

[10] G. Zeng, Y. He, D. D. Ma, S. Luo, S. Zhou, C. Cao, X. Li, X. T. Wu, H. G. Liao, Q. L. Zhu, *Adv. Funct. Mater.* **2022**, 32, 2201125.

[11] W. Zhang, S. Yang, M. Jiang, Y. Hu, C. Hu, X. Zhang, Z. Jin, *Nano Lett.* **2021**, 21, 2650.

[12] P. Lamagni, M. Miola, J. Catalano, M. S. Hvid, M. A. H. Mamakhel, M. Christensen, M. R. Madsen, H. S. Jeppesen, X. M. Hu, K. Daasbjerg, *Adv. Funct. Mater.* **2020**, 30, 1910408.

[13] G. Jia, Y. Wang, M. Sun, H. Zhang, L. Li, Y. Shi, L. Zhang, X. Cui, T. W. B. Lo, B. Huang, *J. Am. Chem. Soc.* **2023**, 145, 14133.

[14] D. Lai, M. Xie, H. Zhao, X. Wu, B. Lv, G. Jing, *Electrochim. Acta* **2023**, 464, 142893.

[15] S. Liu, Y. Fan, Y. Wang, S. Jin, M. Hou, W. Zeng, K. Li, T. Jiang, L. Qin, Z. Yan, *Nano Lett.* **2022**, 22, 9107.

[16] J. H. Koh, D. H. Won, T. Eom, N.-K. Kim, K. D. Jung, H. Kim, Y. J. Hwang, B. K. Min, *ACS Catal.* **2017**, 7, 5071.

[17] Q. Gong, P. Ding, M. Xu, X. Zhu, M. Wang, J. Deng, Q. Ma, N. Han, Y. Zhu, J. Lu, *Nat. Commun.* **2019**, 10, 2807.

[18] D. Wu, X. Wang, X.-Z. Fu, J.-L. Luo, *Appl. Catal. B* **2021**, 284, 119723.

[19] M. Zhang, W. Wei, S. Zhou, D.-D. Ma, A. Cao, X.-T. Wu, Q.-L. Zhu, *Energy Environ. Sci.* **2021**, 14, 4998.

[20] B. Wulan, L. Zhao, D. Tan, X. Cao, J. Ma, J. Zhang, *Adv. Energy Mater.* **2022**, 12, 2103960.

[21] Y. Wang, B. Wang, W. Jiang, Z. Liu, J. Zhang, L. Gao, W. Yao, *Nano Res.* **2022**, 1.

[22] S.-F. Tang, X.-L. Lu, C. Zhang, Z.-W. Wei, R. Si, T.-B. Lu, *Sci. Bull.* **2021**, 66, 1533.

[23] Y. Zhang, X. Zhang, Y. Ling, F. Li, A. M. Bond, J. Zhang, *Angew. Chem. Int. Ed.* **2018**, 57, 13283.

[24] X. Chen, J. Chen, H. Chen, Q. Zhang, J. Li, J. Cui, Y. Sun, D. Wang, J. Ye, L. Liu, *Nat. Commun.* **2023**, 14, 751.

[25] S. Yang, H. An, S. Arnouts, H. Wang, X. Yu, J. de Ruiter, S. Bals, T. Altantzis, B. M. Weckhuysen, W. van der Stam, *Nat. Catal.* **2023**, 6, 796.

[26] C. W. Lee, J. S. Hong, K. D. Yang, K. Jin, J. H. Lee, H.-Y. Ahn, H. Seo, N.-E. Sung, K. T. Nam, *ACS Catal.* **2018**, 8, 931.

[27] T. Tran‐Phu, R. Daiyan, Z. Fusco, Z. Ma, R. Amal, A. Tricoli, *Adv. Funct. Mater.* **2020**, 30, 1906478.

[28] P. Deng, F. Yang, Z. Wang, S. Chen, Y. Zhou, S. Zaman, B. Y. Xia, *Angew. Chem. Int. Ed.* **2020**, 59, 10807.

[29] Y. Xu, Y. Guo, Y. Sheng, H. Yu, K. Deng, Z. Wang, X. Li, H. Wang, L. Wang, *Small* **2023**, 19, 2300001.

[30] S. Liu, X. F. Lu, J. Xiao, X. Wang, X. W. Lou, *Angew. Chem. Int. Ed.* **2019**, 58, 13828.

[31] D. Wu, G. Huo, W. Chen, X.-Z. Fu, J.-L. Luo, *Appl. Catal. B* **2020**, 271, 118957.

[32] P. Deng, H. Wang, R. Qi, J. Zhu, S. Chen, F. Yang, L. Zhou, K. Qi, H. Liu, B. Y. Xia, *ACS Catal.* **2019**, 10, 743.

[33] C. Li, Z. Liu, X. Zhou, L. Zhang, Z. Fu, Y. Wu, X. Lv, G. Zheng, H. Chen, *Energy Environ. Sci.* **2023**, 16, 3885.

[34] Y. Zhang, F. Li, X. Zhang, T. Williams, C. D. Easton, A. M. Bond, J. Zhang, *J. Mater. Chem. A* **2018**, 6, 4714.

[35] F. Li, G. H. Gu, C. Choi, P. Kolla, S. Hong, T.-S. Wu, Y.-L. Soo, J. Masa, S. Mukerjee, Y. Jung, J. Qiu, Z. Sun, *Appl. Catal. B-Environ.* **2020**, 277, 119241.

[36] M. Liu, Y. Wang, T. Yu, L. Zhan, X. Zhao, C. Lian, Y. Xiong, X. Xiong, Y. Lei, *Sci. Bull.* **2023**, 68, 1238.

[37] B. Q. Tian, J. J. Hou, T. Wang, Y. Gao, J. Zhang, W. Lu, J. Jia, *ChemCatChem* **2024**, 16, e202401007.

[38] J. Chen, T. Mao, J. Wang, J. Wang, S. Wang, H. Jin, *Angew. Chem. Int. Ed.* **2024**, 63, e202408849.
